# The role of water insecurity in influencing water and sugar-sweetened beverage choices: A scoping review

**DOI:** 10.1371/journal.pstr.0000174

**Published:** 2025-05-12

**Authors:** Laina Ewoldt, Ana Clara Duran, Checkna Diawara, Carolina Batis, Deshira D. Wallace, Paul Taillie, Joshua D. Miller, Shu Wen Ng, Ryan Cronk, Lindsey Smith Taillie

**Affiliations:** 1 Department of Nutrition, Gillings School of Global Public Health, University of North Carolina at Chapel Hill, Chapel Hill, North Carolina, United States of America; 2 Center for Food Studies and Research (NEPA), University of Campinas, Campinas, São Paulo, Brazil; 3 Center for Epidemiological Studies in Health and Nutrition (NUPENS), School of Public Health, University of São Paulo, São Paulo, São Paulo, Brazil; 4 Center for Research on Nutrition and Health, National Institute of Public Health, Cuernavaca, Morelos, Mexico; 5 Department of Health Behavior, Gillings School of Global Public Health, University of North Carolina at Chapel Hill, Chapel Hill, North Carolina, United States of America; 6 Department of Geography and Environment, University of North Carolina at Chapel Hill, Chapel Hill, North Carolina, United States of America; 7 Carolina Population Center, University of North Carolina at Chapel Hill, Chapel Hill, North Carolina, United States of America; 8 The Water Institute, Gillings School of Global Public Health, The University of North Carolina at Chapel Hill, Chapel Hill, North Carolina, United States of America

## Abstract

Water is a critical nutrient for human health, however more than 4 billion people globally lack access to safe drinking water and climate change is expected to worsen water insecurity. Simultaneously, consumption of packaged water and sugar-sweetened beverages (SSBs) is increasing globally. Despite many plausible linkages, little is known about the relationship between water insecurity and sugar-sweetened or packaged beverage selection. The current study aimed to characterize the relationship between water insecurity and beverage selection by conducting a scoping review to identify trends in available research on beverage selection among individuals experiencing water insecurity, and creating a conceptual model explaining this relationship. The Preferred Reporting Items for Systematic reviews and Meta-Analyses extension for Scoping Reviews (PRISMA-ScR) guided the systematic search of 4 databases, which resulted in the inclusion of 82 studies from 7 geographical regions, representing both middle- and high- income countries. Key emergent themes included perceptions of non-packaged water characteristics, adaptive behaviors, and how each alter consumer selection of packaged water and SSBs. Frequently mentioned non-packaged water characteristics included perceived safety (n = 49; 60%), taste (n = 31; 38%), convenience/accessibility (n = 29; 35%), cost (n = 18; 22%), appearance/turbidity (n = 12; 15%), smell (n = 10; 12%), temperature (n = 9; 11%), and hardness (n = 5; 6%). Reported adaptive strategies included water treatment/filtering (n = 25; 30%) and water testing (n = 5; 6%). Associations between water insecurity and non-packaged water, packaged water, and SSB selection varied by country income classification and demographic characteristics. These can inform potential areas for future interventional trials aiming to increase trust in and selection of plain water as well as reduce reliance on packaged or sugar-sweetened beverages.

## Author summary

Water insecurity is a critical barrier for meeting nutritional needs. When people do not have sufficient access to non-packaged water (such as tap, well, or rainwater) that they deem acceptable for use, they may select alternative beverages for hydration. Alternatives include packaged beverages and sugary drinks, which can harm the environment through plastic pollution and human health through increased intake of added sugars. We searched the available research to identify factors that influence decisions about drinking non-packaged water and other beverages. Available research suggests that consumers identify water characteristics, such as perceived safety, taste, and temperature, as being critical in their decision about whether to use non-packaged water. When these characteristics were unfavorable, many consumers selected packaged water or sugary drinks instead. Typically, individuals who reported using water treatment methods or testing their water safety were more likely to use non-packaged water. These modifiable risk factors can be addressed through policy and programmatic interventions to improve trust in and use of non-packaged water as well as reduce intake of packaged water or sugary drinks.

## 1. Introduction

Maintaining euhydration through fluid replenishment is essential for supporting activities that are critical for human health, including cellular homeostasis, thermoregulation, and physical and cognitive function [1]. Water is a critical source of hydration, yet an estimated 4.4 billion people globally do not have access to safely managed drinking water sources [2]. Access is, however, only one aspect of water insecurity, which also includes aspects of water availability, acceptability and sufficiency of water for diverse household uses, and reliability across time [3]. Climate change is expected to compound existing water issues by shifting precipitation patterns, intensifying freshwater salinization, and increasing the frequency of floods, droughts, and heat waves, especially in low-resource settings [4,5]. Climate-induced shocks are projected to not only contaminate fresh-water sources [4], but also impact water insecurity through damage to water storage, treatment, and delivery systems [6,7]. Concurrently, a growing global population and increasing temperatures will increase global freshwater needs [8]. Individuals with outdoor or physically intensive occupations may be at heightened risk of dehydration since water demands increase in warmer environments [9]. As a result, it is critical to understand how beverage selection is shifting or may shift in the future in the context of climate and environmental changes.

Shifts in beverage selection due to factors other than climate and environmental changes have already been occurring globally. Consumption of packaged water and sugar-sweetened beverages (SSBs) is increasing globally, in part due to aggressive marketing tactics and distrust of water safety in both high- and low-income countries [10–13]. There is concern that climate change-induced water insecurity may exacerbate this trend, with individuals substituting non-packaged water with beverages that are less optimal for human and planetary health. Both packaged water and SSBs contribute to environmental harm in the form of plastic pollution and groundwater depletion [13–16]. Furthermore, although packaged water is typically perceived as safe by consumers, consuming beverages from plastic bottles may also harm human health through increasing intake of microplastics and exposing consumers to contaminated water in the case of inadequate treatment practices or poor storage techniques [17,18]. Additionally, in many regions, SSBs may be preferred over water due to availability, cost, convenience, or taste, which is concerning due to their association with increased risk of chronic diseases, including obesity, type 2 diabetes, cardiovascular disease, poor dental outcomes, and kidney disease [19–22].

Considering the health concerns associated with increased consumption of packaged beverages, it is important to better understand how increases in water insecurity may affect this behavior. Despite many plausible linkages, little is known about the relationship between water insecurity and non-packaged water, packaged water, and SSB selection. Although several studies have assessed consumer preferences surrounding non-packaged water, packaged water, and SSBs, there is a paucity of research systematically compiling these findings and the relationships between them. Understanding what is known about how water insecurity alters consumers’ beverage behaviors is important for designing interventions and policies that minimize its negative impacts on planetary and human health.

The current study aimed to address this through two objectives. The first aim was to conduct a scoping review to identify trends in available research on beverage selection by water insecurity status and to describe any variability in this relationship by geography or other population characteristics. The second aim was to create a conceptual model using findings from the scoping review to describe the pathways between water insecurity and beverage selection. Together, this information can help inform future research and ultimately the design of effective interventions.

## 2. Methods

### 2.1. Protocol and registration

The review protocol was developed using the Preferred Reporting Items for Systematic reviews and Meta-Analyses extension for Scoping Reviews (PRISMA-ScR) [23]. It was registered at the Open Science Framework on October 30, 2023 (https://osf.io/nbjyz).

### 2.2. Eligibility criteria

Study eligibility criteria were drafted through team discussion (Table 1). Only human studies were included in the review. There were no restrictions for participant age, publication date, or setting. Observational studies needed to include a domain of water insecurity (availability, accessibility, acceptability or sufficiency for use, or stability across time) as the exposure, and interventional studies needed to intervene upon a water insecurity domain. Outcomes of interest included the availability, purchase, or intake of any beverage other than water from an improved water source (excluding packaged water) [24], which will be referred to as non-packaged water (NPW) for the reminder of the review. This definition includes piped water, boreholes/tubewells, protected dug wells, protected springs, and rainwater [24]. Beverages selected instead of NPW, referred to as “alternative beverages” throughout the review, included packaged water and SSBs such as soda, sports drinks, juice, or sweetened dairy beverages. Alcoholic beverages were not included, as they are not primarily consumed for the purpose of hydration. Quantitative, qualitative, and mixed methods studies were included to identify measurable and perceived drivers and consequences of water insecurity. Reviews were not included to avoid overrepresenting studies that may have been included in multiple reviews. Finally, studies were only included if they were written in the authors’ first language: English, Portuguese, or Spanish.

### 2.3. Search strategy and sources

The study team developed a systematic search strategy with the assistance of a health sciences librarian. The following terms terms were search in PubMed: (beverage[tiab] OR soda[tiab] OR “carbonated beverage”[tiab] OR “carbonated beverages”[tiab] OR “fizzy drink”[tiab] OR “fizzy drinks”[tiab] OR pop[tiab] OR “sugar-sweetened beverage”[tiab] OR “sugar-sweetened beverages”[tiab] OR (sugar[tiab] AND beverages[tiab]) OR “sugared beverages”[tiab] OR (sweetened[tiab] AND beverages[tiab]) OR “sweet drinks”[tiab] OR “sweetened drinks”[tiab] OR “sugary drinks”[tiab] OR juice[tiab] OR juices[tiab] OR nectar[tiab] OR nectars[tiab] OR “fruit drink”[tiab] OR “fruit drinks”[tiab] OR “fruit flavored beverage”[tiab] OR “fruit flavored beverages”[tiab] OR “soft drinks”[tiab] OR “soft drink”[tiab] OR “energy drink”[tiab] OR “energy drinks”[tiab] OR coffee[tiab] OR tea[tiab] OR “sports beverage”[tiab] OR “sports beverages”[tiab] OR “sports drink”[tiab] OR “sports drinks”[tiab] OR “bottled water”[tiab]) AND (“Water”[Mesh] OR “Water Resources”[Mesh] OR “Water Supply”[Mesh] OR water[tiab] OR h2o[tiab]) AND (Availability[tiab] OR access[tiab] OR supply[tiab] OR intake[tiab] OR security[tiab] OR insecurity[tiab] OR shortage[tiab] OR unavailable[tiab] OR scarcity). Language and human studies filters were applied to narrow the search.

The Polyglot tool from The Systematic Review Accelerator was used to convert these search terms for use on Scopus, Global Health, and Embase. Additional studies were identified by manually scanning reference lists of included studies. Search results were imported into EndNote for further refinement, and then Covidence for storage and review. The last search was conducted on November 3, 2023.

### 2.4. Study selection

After the search was conducted, two authors individually screened studies to determine whether they met the inclusion criteria. A third author resolved any discrepancy in opinion. This screening process occurred in two stages, first with titles and abstracts and second with whole texts. Screening was conducted by LE, CB, LST, and a team of research assistants.

### 2.5. Data extraction

The lead author (LE) systematically extracted the following information from each included study: study name, authors, publication year, study design, setting, study aim(s), population, unit of analysis, study size, exposure/intervention, baseline population characteristics, and beverage outcomes. The study team developed a data extraction form to standardize this process.

### 2.6. Synthesis of results

Inductive coding was used to identify themes and categorize the extracted data. The following themes were identified by LE during review of the extracted data: characteristics affecting NPW selection, packaged water as an alternative beverage, sugar sweetened beverages as an alternative beverage, NPW modifications affecting selection, and sociodemographic differences in alternative beverage choices. Data extracted from the initial review was then organized into these identified categories. Differences by HICs and LMICs, determined by World Bank income classification (fiscal year 2024), were examined within these categories.

### 2.7. Conceptual model

Study authors initially drafted a conceptual model that identified potential pathways between climate change and human health. To reflect the complex and multi-directional nature of these relationships, the interdisciplinary team of study authors contributed expertise spanning the fields of nutrition, epidemiology, health behavior, geography, environmental science, economics, and water. This model identified water insecurity and beverage selection as a critical nexus where additional research is needed. This informed the decision to conduct a scoping review focused on how water insecurity impacts selection of packaged water and SSBs. After the scoping review was conducted, a new conceptual model was created that focused on water insecurity and beverage selection, incorporating the scoping review findings about the water characteristics driving this relationship.

## 3. Results

### 3.1. Scoping review

**Fig 1.**
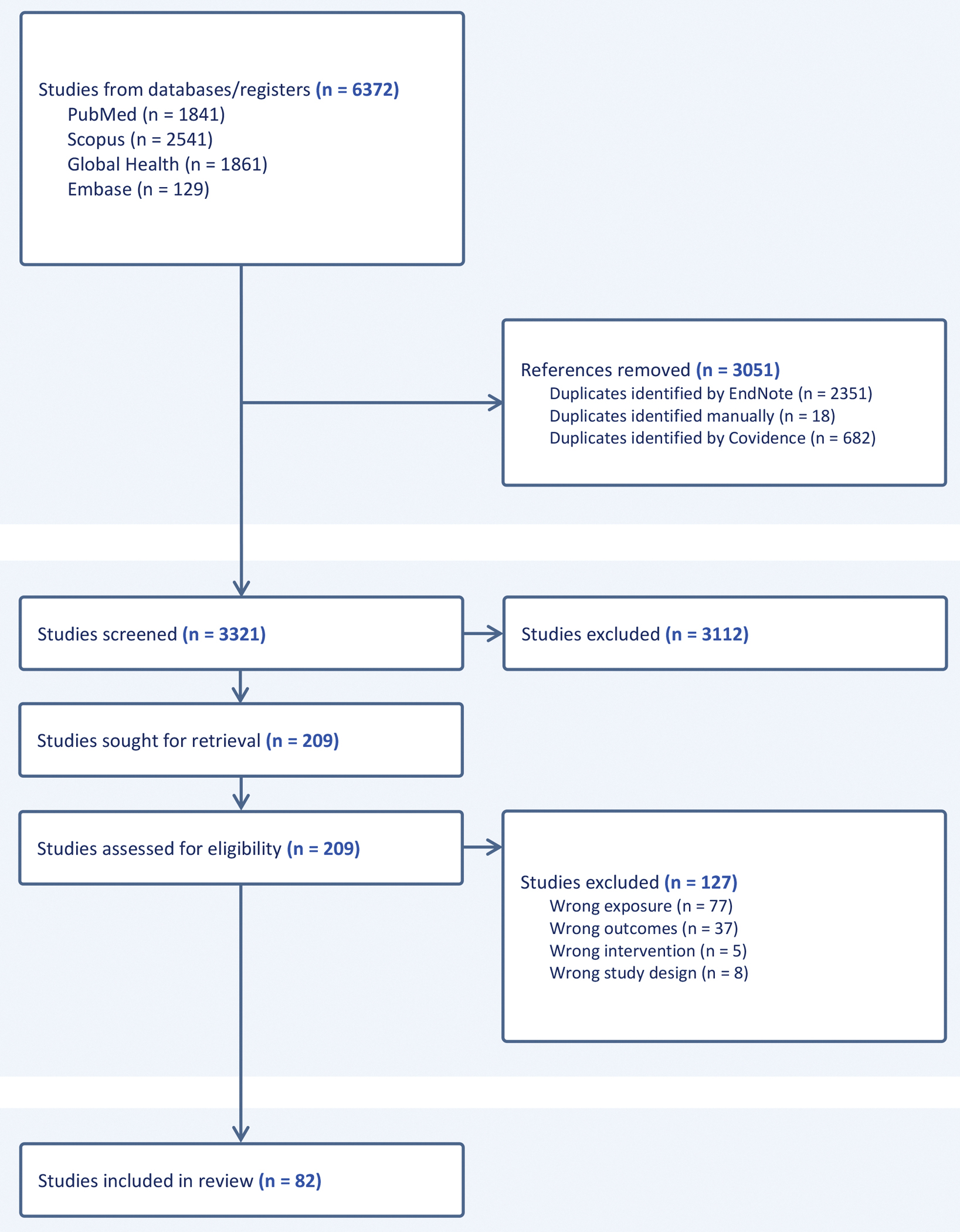
PRISMA diagram for a scoping review on water insecurity and alternative beverage choices.

The initial search returned 6732 studies. Endnote and Covidence removed 3051 duplicates, leaving 3321 studies to screen. 3112 studies were excluded based on title and abstract review. Of the 209 remaining studies, 82 were selected for inclusion following full-text review (Fig 1).

### 3.2. Study characteristics

**Fig 2.**
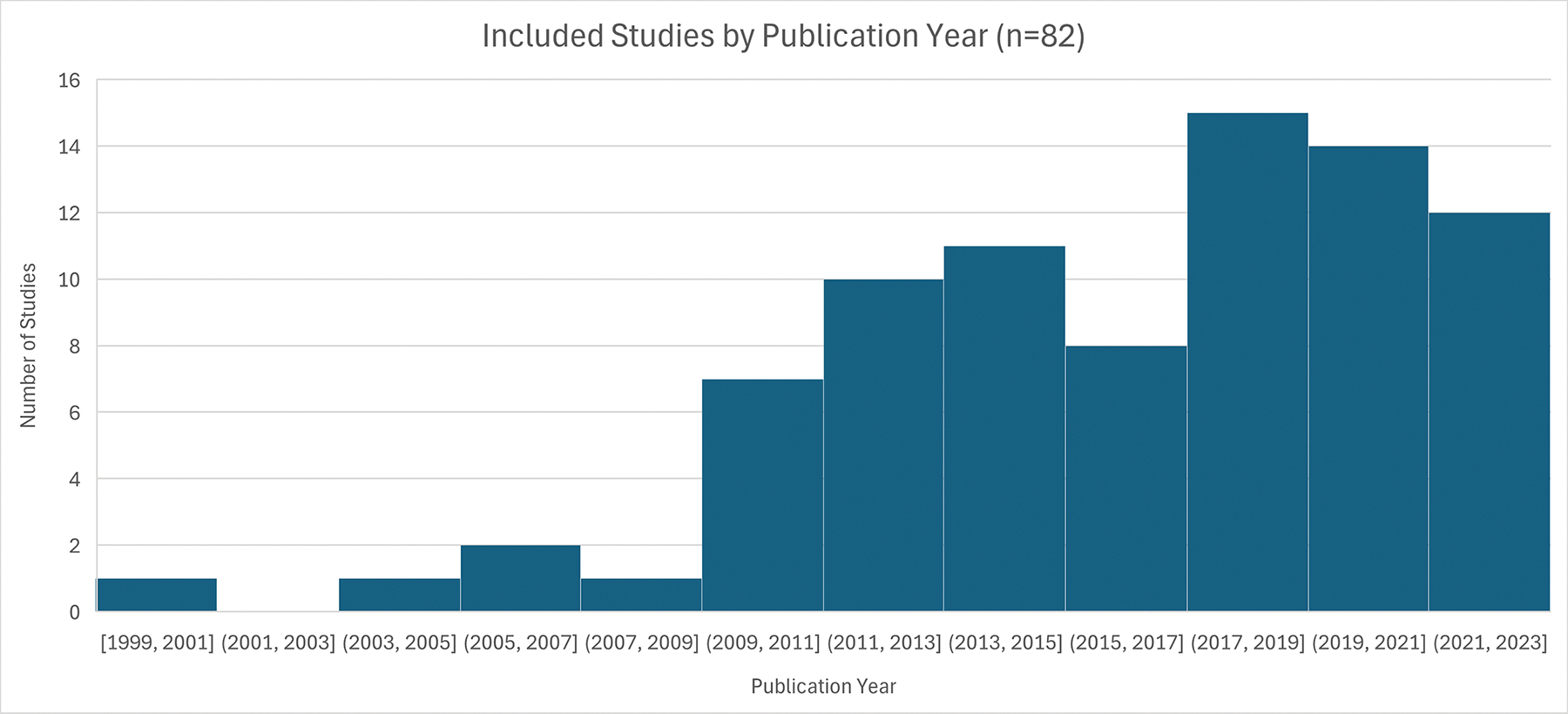
Histogram of publication year of studies in a scoping review on water insecurity and alternative beverage selection.

In general, we observed a greater number of published studies focusing on this topic across time, with 67% of the studies published since 2015 compared to only 33% between 1999 and 2014. (Fig 2).

Sixty-six percent of studies were conducted in North America (n = 54), 12% were conducted in Europe (n = 10), and the remaining 12% percent were distributed across other geographical regions listed in Table 2. More studies were conducted in HICs (n = 60), with less than a third of the studies including LMICs (n = 26). The included studies were primarily observational in design (n = 67), thirty-seven of which were cross-sectional. Fifteen interventional studies were included, which sought to increase water access or quality and typically focused on children or families with young children. Regarding sociodemographic characteristics, age and gender were the most commonly reported (n = 49 both), followed by education (n = 34), income (n = 31), and race/ethnicity (n = 28).

### 3.3. Water insecurity and alternative beverage choices

Of the included studies, 65 (79%) assessed packaged water selection when a domain of water insecurity (e.g., availability) was suboptimal and 56 (68%) reported increased packaged water use. Forty-one studies (50%) investigated the relationship between water insecurity and SSB selection, of which 33 (40%) reported increased utilization when a domain of water insecurity was suboptimal. While two studies provided estimates of the association between water insecurity and alternative beverage selection [25,26], most assessed the relationship with specific water characteristics. For example, a survey of adults in the United States (US) adults found that tap water avoidance (a proxy for water insecurity, 0 mL/day tap water intake) was associated with 620 mL/day higher bottled water intake (95% CI: 570, 669) and 24% higher odds of consuming >10% of total daily kcals from added sugar from SSBs (95% CI: 1.07, 1.43) [25]. Conversely, a cross-sectional study of low-income elementary school students in the US reported reduced frequency of fountain water intake among students experiencing water insecurity (RD: −0.5 times per day; 95% CI: −0.8, −0.3), but observed no difference in bottled water intake (0.5 times per day in both groups) [26].

### 3.4. NPW characteristics affecting beverage selection

**Fig 3.**
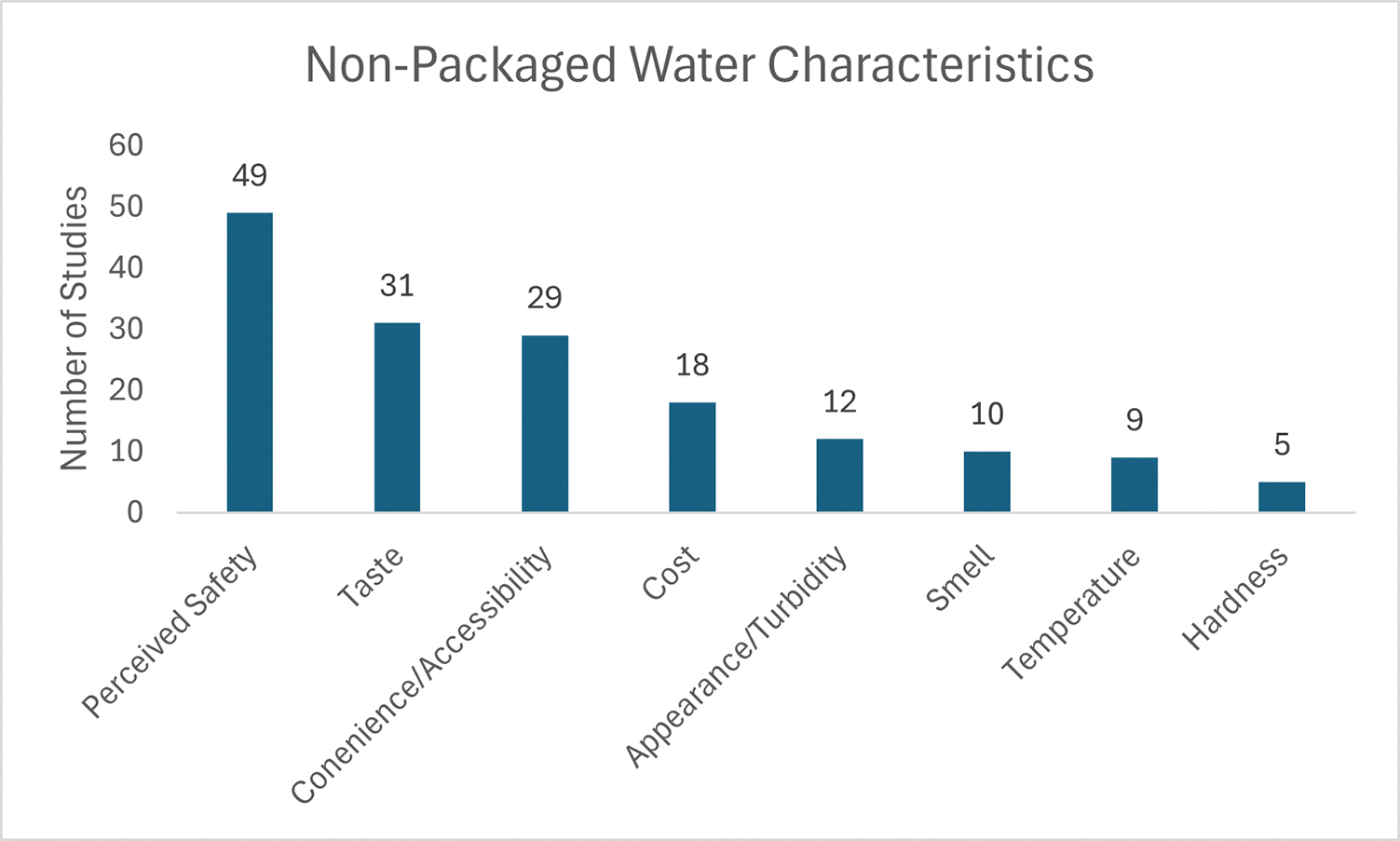
Count of studies reporting non-packaged water characteristics affecting beverage selection in a scoping review on water insecurity and alternative beverage selection (n = 82).

The scoping review revealed 8 water characteristics that influence NPW selection (Fig 3): perceived safety (n = 49; 60%), taste (n = 31; 38%), convenience/accessibility (n = 29; 35%), cost (n = 18; 22%), appearance/turbidity (n = 12; 15%), smell (n = 10; 12%), temperature (n = 9; 11%), and hardness (n = 5; 6%).

#### 3.4.1. Perceived safety.

Perceived safety of NPW was most commonly identified as a factor impacting NPW selection (n = 49; 60% of total studies) [27–75]. Among these studies, sixteen (33%) took place in LMIC settings and 36 (73%) in HIC settings. Other NPW characteristics such as taste, smell, or appearance (i.e., the organoleptic properties of water), influenced consumers’ perceptions of water safety [45,52,71]. Interestingly, chlorine, a common water treatment option, led some to view water as unsafe due to its odor [52]. Among the studies that specified why water was viewed as unsafe, reported concerns included source concerns [52,66,69,72], old/faulty infrastructure [61,71], a history of poor water quality or health department warnings [32,33,60,71], and documented or perceived contamination with chemicals or [36,45,47,56,57,60,67] microorganisms/pathogens [36,47,49,52,55,56,67] as well as the presence of heavy metals [36,43,47,53,55,57,69,74] fluoride [61], or agricultural contamination/nitrate compounds [35,47,67,71]. In six studies, some respondents attributed prior illnesses to the quality of NPW [48–50,54,71,75]. Some studies found that respondents considered NPW at school or other settings outside the home to be less safe than NPW available at home [28,64].

Concerns about water safety discouraged individuals from drinking NPW, although some studies reported that even when water was perceived to be unsafe, individuals may still use it for cooking [42,43,63,69,71,76], washing produce [42,63,69], and cleaning or sanitation [42,69,76]. Cooking may reduce levels of some contaminants, however, use of unsafe water for food or beverage preparation can still result in exposure to harmful substances [77].

##### Alternative Beverages:

Twenty-three studies (28% of total studies) reported how perceived NPW safety influenced alternative beverage choice, with 16 examining packaged water and 10 examining SSBs [27–30,32,34,35,37,38,41,44,47–50,52,59,65,66,70,71,78,79]. Eight studies reported the percentage of study participants who reported that packaged water is safer or healthier than NPW, with responses ranging from 26% to 100% [29,30,37,47,48,50,52,70]. In a survey of 5,823 US adults, respondents who did not view home tap water as safe were 5.9 times as likely to exclusively use bottled water (OR: 5.88; 95% CI: 4.46, 7.76) and 1.7 times more likely to use bottled water regularly (OR: 1.74; 95% CI: 1.31, 2.32) [34]. In rural Saskatchewan, households had higher odds of using bottled water as their primary water source if they had received a prior water advisory compared to those without a prior advisory (OR: 1.7; 95% CI: 1.3, 2.4). Additionally there was a larger magnitude of association among those who had no aesthetic water complaint (OR: 2.3; 95% CI: 1.4, 3.8) compared to those with an aesthetic water complaint (OR: 8.5; 95% CI: 5.2, 13.9) [32].

Despite its poor nutritional composition, consumers may believe SSBs are a healthier beverage for maintaining hydration than water when available water is considered unsafe [59,66,71]. In one qualitative study, Latino parents in the US reported that although they believe water is the healthiest beverage for their children, many will provide SSBs such as soda, sports drink, or juice if bottled or filtered water are unavailable [71]. A study of Michigan adults before and after the Flint Water Crisis found that adults living in Flint, Michigan had higher odds of soda (1.24; 95% CI: 1.01, 1.45), fruit juice (1.55; 95% CI: 1.14, 2.11), and other sugary drink (1.44; 95% CI: 1.04, 2.01) intake after the crisis compared to before [79].

#### 3.4.2. Taste.

Taste preference was the second most frequently mentioned driver of NPW selection (n = 31; 38% of total studies) [31,37–40,45–47,49,51,52,56–63,65–67,69–74,76,80,81]. Of studies addressing taste, nine (29%) included LMICs and twenty-four (77%) included HICs. Of the studies that reported a specific disliked flavor, five mentioned chlorine taste [39,59,63,69,72], four mentioned mineral or metallic taste [45,59,63,74], and two mentioned salty water [69,76]. Some studies found that taste was a more salient motivating factor for beverage selection than perceived safety [37,40,46,51,70]. Taste and perceived safety may be linked, with one study reporting a positive association between water taste preferences and safety concerns (Standard β: 0.444; p < 0.001) [65]. Notably, taste as a driving factor for beverage selection ranked lower for primary NPW drinkers than for primary packaged water drinkers [65,80].

##### Alternative Beverages:

Fifteen studies (18% of total studies) examined how NPW taste impacted alternative beverage selection, 14 of which included packaged water and two of which included SSBs [37–39,47,49,51,52,56,61,65–67,70,71,80]. Disliking the taste of NPW increased the likelihood of using packaged water [47,49,56,61]. Seven studies found a preference for the taste of packaged water over NPW [39,51,52,61,67,70,71]. In two studies, consumers considered taste a stronger motivator for home packaged water selection than for packaged water selection away from the home [39,56]. Among US adults, Park et al. (2023) found that those who believed their tap does not taste good had nearly 3 times the odds of drinking more than one cup per day of bottled water (OR: 2.91; 95% CI: 2.39, 3.53) compared to those who believed their tap water tasted good, although there was no association with SSB intake [37]. Interestingly, one study of Canadian Indigenous communities revealed that some households add sugary drink mix or juices to improve the taste of NPW perceived to be of poor quality [66].

#### 3.4.3. Convenience/Accessibility.

Twenty-nine studies (35% of total studies) mentioned the accessibility or convenience of NPW relative to other beverages [31,39,51–56,58–61,65–70,72,73,75,76,80–86]. Of studies addressing convenience or accessibility, twelve (49%) included LMICs and eighteen (62%) included HICs. In some settings, consumers valued NPW for its accessibility or convenience [54,60,61,70,72,80]. In Thailand, Hill Tribe members who used mountain water or shallow wells, valued for their year-round sufficiency, prioritized convenience when choosing a water source more than those who used bottled water [54]. In Argentina, 60.2% of adults surveyed by Fortunato et al. (2020) listed access to tap water at home as their primary reason for not drinking bottled water [70], and in Los Cabos, Mexico, households reported a high level of satisfaction with water being available day and night due to new desalinization plants, despite lingering concerns about the tap water quality [69].

Nonetheless, NPW was not sufficiently accessible in the settings of all studies included in this scoping review [39,52,55,59,65,68,73,75]. Schools commonly reported issues with water accessibility, with several studies reporting that schools had insufficient, broken, or poorly dispersed drinking water systems [31,39,58,59,73,81,83]. Two studies, both in LMICs, noted seasonal variability in water accessibility [76,85]. Rural communities may have additional problems. For instance, in 2015, Canadian Indigenous households in Black Tickle-Domino were located 1–2 kilometers away from the potable drinking water unit [66]. High transportation costs, especially during inclement weather, and frequent closures due to insufficient funding, prevented many households from utilizing the potable drinking water [66].

##### Alternative Beverages:

Twenty-eight studies (34% of total studies) reported how NPW convenience or accessibility impacted alternative beverages, with 15 including packaged water and 19 including SSBs [39,51–53,58–60,65,67,71,72,75,76,80–83,85–95]. Some studies found that perceived packaged water convenience, particularly when away from the home, was a key determinant of use [51,52,59,67,71,72,80,90]. In one qualitative study, parents of middle and high school students living in the rural South-western US explained that bottled water is more convenient than tap water because it provides reliable water access even if students are in an area without safe tap water [59]. One study among Alaskan adults found that household without access to piped water had 29% higher odds of SSB intake in households (OR: 1.29; 95% CI: 1.00, 1.67) compared to those with reliable access [78].

Numerous interventions included in the scoping review sought to increase NPW selection and decrease SSB or packaged water selection by improving the convenience and accessibility of NPW in schools, households, and communities [53,81,86–89,91–97]. These interventions resulted in null to small reductions in SSB selection. A pilot study that provided filtered tap water dispensers and promotional activities in a US middle school observed an increase in tap water intake, but no change in bottled water or SSB intake, with 29% of students interviewed post-intervention saying that they preferred bottled water to tap water [87]. Similarly, a study that installed new bottled water filling stations in Philadelphia recreation centers observed no change in the average number of youth bringing SSBs or bottled waters, despite an average adjusted increase in tap water usage of 8.6 gallons per day per intervention site compared to the control sites (Difference in differences (DID): 8.6; 95% CI: 4.2, 13.0) [88]. Among intervention center staff however, there was a 34.8 count reduction in the mean frequency of SSBs consumed over the past thirty days compared to the control center staff (DID: −34.8; 95% CI: −67.7, −1.9) [88].

#### 3.4.4. Cost.

Cost was the fourth most frequently mentioned consideration when evaluating NPW selection (n = 18; 22% of total studies) [52,60–73,76,80,81]. Typically, consumers considered NPW to be more affordable [60,61,63,65,69,72,80]. Of studies addressing cost, seven (39%) included LMICs and eleven (61%) included HICs. Similarly, respondents in other studies reported an aversion to the cost of packaged water [60,64,67,68,70,73,76,81], with two exceptions. Several community members in Black Tickle-Domino considered the 2 Canadian dollar per liter cost of obtaining water from the potable drinking water unit too high [66]. Additionally, residents in Turbo, Antioquia slums reported that high fuel costs limited their ability to boil water [76]. In both studies, however, packaged water was also considered expensive. The primary concern mentioned about NPW affordability arose at an institutional level, where the cost of updating, repairing, and maintaining community or school water systems may be prohibitively expensive [53,66,73].

##### Alternative Beverages:

Eleven studies (13% of total studies) reported data on NPW cost in relation to alternative beverages, with 10 including packaged water and 2 including SSBs [46,52,55,59,64,65,71,73,76,81,90]. Scherzer et al. (2010) found that participants in a rural California community did not want to purchase bottled water or water mill water if their tap was proven to be safe [71], and Larson et al. (1999) found that more survey respondents living in Moscow opted for “no-cost” water options such as boiling (88%) or settling (33%) tap water instead of purchasing bottled water (13%) [46]. Although consumers often consider affordability an attractive aspect of NPW relative to alternative beverages, several studies have noted how concerns about other NPW characteristics (e.g., perceived safety) have a larger impact on behavior [64,67,73,81]. Furthermore, the low cost of NPW may increase the perceived value of packaged water; four studies (three in LMICs, one in a HIC) documented that selecting packaged water instead of NPW conferred higher social status [52,64,65,90].

The studies that considered SSBs highlighted that free NPW has a higher probability of selection over SSBs than purchasable water alternatives [59,73]. In a study of California middle schools, some school stakeholders expressed concerns that offering free NPW could negatively affect USDA meal reimbursements, vendor contracts, or funding for extracurricular activities supported by alternative beverage sales [73]. Beyond NPW costs, it is also important to note that some studies documented lower costs of SSBs that packaged water for some water-insecure individuals, in which study respondents indicated that SSBs would be preferentially purchased [31,45,57,59,62,66]. In one US study, a participant explained that the Special Supplemental Nutrition Program for Women, Infants, and Children requirements contributed to this discrepancy in affordability, as benefits covered the cost of fruit juice but not bottled water [45].

#### 3.4.5. Appearance/Turbidity.

In the 12 studies (15% of total studies) that mentioned the appearance or turbidity of water, consumers universally preferred clear and colorless water [40,45,47,49,52,54,57,62,66,71,73,76]. Of the studies addressing appearance or turbidity, three (25%) included LMICs and nine (75%) included HICs. Naturally occurring minerals or aging infrastructure can cause water to appear yellow, orange, brown, or “rusty” [45,49,57,62,66,71]. Cloudy or turbid water decreased acceptability of NPW [40,45–47,54,76]. Jones et al. (2006) reported that respondents commonly treated their water to reduce cloudiness [47]. Visibly dirty or unclean water sources may also discourage usage [66,73].

Only two studies provided information about the relationship between the appearance or turbidity of NPW and alternative beverages. In one study, bottled water drinkers ranked reduced cloudiness as an important characteristic of bottled water [47]. In another study, respondents mentioned powdered drink mix as a method to mask off-colors in NPW [66].

#### 3.4.6. Smell.

Smell, which is closely related to taste, was explicitly mentioned in fewer studies (n = 10; 12% of total studies) [45–47,49,52,54,56,67,71,76]. Of the studies addressing smell, five (50%) included LMICs and five (50%) included HICs. Similar to taste, chlorine was the most commonly specified negative odor [45,52,71]. Odor alone was the primary water selection criterion for the Yao people in Thailand [54]. In a study of bottled water users in Iran, 95% reported that they preferentially chose bottled water because tap water was believed to have a “disgusting odor” [67].

#### 3.4.7. Temperature.

In the nine studies (11% of total studies) that discussed temperature [31,57–59,64,72–74,81], consumers favorably viewed cold NPW and warm NPW unfavorably. Of the studies addressing temperature, two studies (22%) occurred in LMIC settings and seven studies (78%) in HIC settings. Especially in warm climates, cold water was considered ideal for satisfying thirst [57,59]. Two studies reported how NPW temperature impacts alternative beverage selection. For example, rural Southwestern US middle school and high schooler stated that cold tap water was preferable than non-chilled soda [59], and Australian Indigenous families were more likely to choose SSBs than warm water [57].

#### 3.4.8. Hardness.

Five studies (6% of total studies) mentioned hardness as a NPW characteristic considered by consumers [46,47,59,67,71]. Of the studies addressing NPW hardness, two (40%) studies were conducted in LMICs and three (60%) in HICs. Beyond simply persuading respondents away from drinking their NPW, hardness also discouraged NPW usage for hygiene and sanitation activities. Respondents reported that they could tell their water was hard water because it left residue on dishes, skin, sinks, and clothing [46,59,71]. When assessing treatment of private water supplies in Canada, Jones et al. (2006) discovered that respondents treating their water used water softeners more commonly than other treatment options [47]. Furthermore, in the one study reporting on the relationship between tap water hardness and alternative beverage selection, Canadian respondents with private water supplies considered bottled water superior to NPW due to its reduced hardness [47].

### 3.5. Adaptive behaviors impacting NPW and alternative beverage selection

Water treatment or filtering (n = 25; 30%) and water testing (n = 5; 6%) were primary adaptive strategies for managing NPW considered to be insufficient.

#### 3.5.1. Treatment/Filtration.

Twenty-five studies (30% of total studies) mentioned treatment or filtering at the community or in-home level as a factor impacting NPW selection, typically positively [32,41–47,52–56,58,63,68–72,76,98–101]. Methods described for treating NPW at a household/individual level included boiling [42,46,54,55,68,69,76], filtering [41,42,44–46,53–56,63,70–72,76,99], settling/sedimentation [46,54,76], and various other treatment methods [32,42,43,47,52,55,68,76,98,100,101]. Some studies noted that filter usage could improve taste [47,62,72]. Lavallee et al. (2021) found that households with private wells reporting a water treatment system had 67% higher odds of consuming water daily than those without a treatment system (OR: 1.67; 95% CI: 1.24, 2.26) [101]. Installation of water treatment or desalinization plants can provide new sources of safe NPW, although multiple studies have found that residents may remain hesitant to accept the safety of the new water source. For instance, Fragkou et al. (2016) reported that residents in Mexico and Chile were uncertain of tap safety after desalination plants were installed, with residents preferring to filter or boil water instead of consume water provided from the desalination plants [69].

##### Alternative Beverages:

Five studies (6% of total studies) assessed the relationship between NPW treatment or filtration and alternative beverage utilization, five of which included packaged water and one SSBs [32,42,98–100]. Decreased packaged water selection was observed in three studies [32,98,99], but participants in two studies reported high packaged water intake despite water treatment [42,100]. In rural Saskatchewan, respondents who did not treat their tap water were 4.6 times more likely to primarily drink bottled water than those who did not (OR: 4.6; 95% CI: 2.9, 7.3) [32]. Rosinger et al. (2018) found that using a home water treatment device was associated with a higher likelihood of US adults consuming tap water and decreased likelihood of consuming bottled water (adjusted OR: 1.21; 95% CI: 1.01, 1.4) [98].

In a randomized control trial that provided low-income Latino parents of infants and toddlers living in the US with water filter pitchers, tap water intake increased and SSB intake decreased among intervention households. SSB intake decreased by 11.2 fluid ounces/d (p < 0.01) for parents and 1.50 fluid ounces/day (p = 0.03) for children in the group that received a water filter pitcher and education, and it decreased by 8.0 fluid ounces/day (p < 0.01) for parents and 1.6 fluid ounces/day (p = 0.02) for children in the group that only received a filter [99]. In the study arm that also received education, parents and children were consuming more water from tap water than from bottled water intake at the end of the intervention [99]. In a follow-up to this study, participants reported that the filter increased the perceived safety of tap water, however, some still did not trust that the filter adequately improved water safety [72].

Although filtering and treating NPW is typically cheaper than purchasing packaged water, it is still important to consider the associated costs. Boiling water incurs fuel costs that may be prohibitive for some [66,76]. Filtration also requires upkeep, such as replacing filters or performing maintenance as needed, and updating infrastructure in schools may be cost prohibitive [46,53,73,74].

#### 3.5.2. Water testing.

Five studies (6% of total studies) mentioned water testing [47,53,71,74,101]. Testing can increase community confidence in the safety of NPW; however, results must be communicated effectively. Residents of a low-income rural California community mentioned that although they would be interested in seeing results of the annual water testing that they were previously unaware of, they would be more convinced if the testing was conducted by a non-governmental third-party organization [71]. During interviews with California school district stakeholders, water quality employees explained that the water is tested and adheres to safety requirements, although school employees and families often consider the water to be unsafe [73].

Two interventions, one in a rural California community and another in Philadelphia community centers [53,74], tested the water prior to implementing interventions to address community concerns about tap water safety. Patel (2019) shared the results of the water testing in several formats, both in person and online [53]. After the intervention, survey respondents suggested that ongoing water testing is important to promoting tap water intake [53]. A study of private-well-using adults in Ontario reported that respondents who tested their water, relative to those who did not, had higher odds of consuming 1250–1500 mL (OR: 2.49; 95% CI: 1.35, 4.61) or 1750–2000 mL (OR: 4.74; 95% CI: 1.48, 15.17) of tap water daily [101].

##### Alternative Beverages:

Three studies (4% of total studies) reported data on NPW testing’s association with alternative beverages, with all three including packaged water [47,53,101] and one including SSBs [53]. In the study of private-well using adults in Ontario, those who did not test their water had higher odds of primarily using bottled water than those who tested their well water (OR = 2.15; 95% CI 1.31–3.55) [101].

As discussed in the perceived safety section, many viewed packaged water as safer than NPW, a factor that respondents in Jones (2006) attributed to “better testing” [47]. In reality, packaged water is insufficiently regulated in some countries, a fact noted by five studies included in the scoping review [52,70,90,100,102]. In Ecuador, Lee et al. (2020) found no reduction in diarrhea rates among bottled water users in their study population and noted that prior studies found high rates of contamination in bottled water in Ecuador [100].

### 3.6. Variation in alternative beverage selection by demographics

Age (n = 23), gender (n = 22), and income (n = 22) were the most examined demographics in relation to water insecurity and alternative beverage selection. Most studies found an inverse relationship between age and alternative beverage selection for both packaged water (50%) [29,34,37,40,48,50,102–104] and SSBs (60%) [78,79,105] in populations experiencing water insecurity. In regard to gender, women were more likely than men to drink packaged water in 39% [34,48,49,56,80,101,102] of studies but less likely to consume SSBs in 75% [79,83,105]. The relationship with packaged water selection by income was inconsistent in HICs, although a positive or null association was seen within all LMICs (n = 5 positive; n = 1 null) [46,64,67,85,102,103]. Differential associations between water insecurity and SSBs selection by income were null in all but one study, which reported a negative association between SSBs and income among Indigenous Australian children [105]. The full tally of studies measuring variations by demographic characteristics can be found in Table 3.

### 3.7. Conceptual model

**Fig 4.**
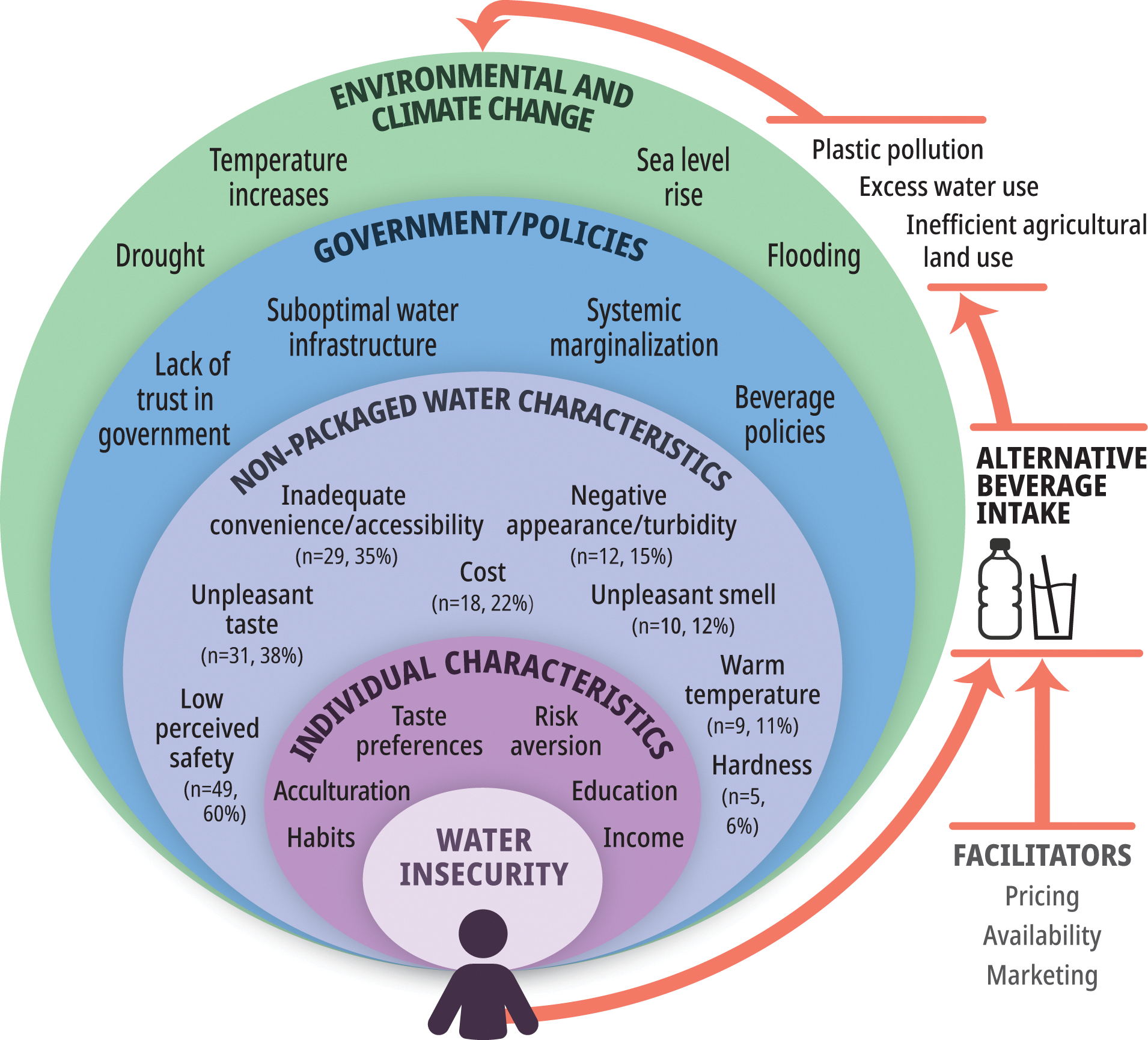
Conceptual model of the relationship between climate change, water insecurity, and the selection of alternative beverages such as packaged water and sugary drinks instead of non-packaged water.

The revised conceptual model (Fig 4) highlights the diverse climate events acting upon water systems, and how factors at each level of the socio-ecological model can alter their impact on water insecurity, and thereby, both NPW and alternative beverage intake. NPW characteristics commonly impacted water insecurity throughout the scoping review, and their interactions with government or policies and individual characteristics highlight the heterogeneity within water insecurity experiences. Finally, the model depicts the bidirectional relationship between alternative beverage intake and climate change. Plastic pollution, excess water usage, and inefficient usage of agricultural land are adverse climate and environmental effects stemming from alternative beverage intake, particularly bottled SSBs [13,106–109].

## 4. Discussion

This scoping review compiled available literature on how water insecurity impacts NPW characteristics and subsequently influences alternative beverage selection, specifically packaged water and SSBs. Eighty-two studies were included, representing countries from all major geographical regions and most World Bank income classifications. Although data on water perceptions are available from a diverse range of countries, studies quantifying the relationship between NPW perceptions and alternative beverage selection primarily occurred in high-income North American countries (US and Canada). This is in contrast to former reviews that focused on water insecurity and coping strategies in general, where the majority of studies were from LMICs [110,111].

Many of the NPW characteristics impacting aspects of water insecurity found in this scoping review (perceived safety, taste, convenience/accessibility, cost, appearance/turbidity, smell, temperature, and hardness) have been mentioned in prior reviews of water insecurity and water preferences [112,113]. This review adds to the literature by documenting the relative frequency with which they are mentioned in water insecurity research and how they relate to alternative beverage selection, although further work is needed to systematically quantify how they impact alternative beverage selection. Many of the relationships discussed relied on qualitative findings, particularly in LMICs. Interventions should target characteristics that have the greatest impact on consumer behavior. As such, understanding the degree to which NPW characteristics influence consumer behavior will help design cost-effective, efficacious interventions that address locally specific water concerns.

Despite the need for additional research prior to large-scale interventions, the relationships between NPW characteristics and alternative beverage use noted in this review can begin to inform which characteristics future interventional trials aiming to decrease alternative beverage selection may need to focus on. For example, if taste is a primary concern, altering treatment methods to achieve a better mineral content may increase selection [114]. In warm climates where colder beverages are especially valued, lowering the temperature of water dispensed from public fountains or recommending residents store water in refrigerated and covered pitchers can increase the perceived value of NPW in comparison to alternative beverages.

The revised conceptual model shows the multilevel factors impacting water insecurity, and the NPW characteristics informed by the scoping review can help identify areas to target future research and where interventions can ameliorate water insecurity and decrease alternative beverage selection. Interventions that will provide long-term solutions to the issue of water insecurity and alternative beverage selection will require additional investment and governmental support. Aging or faulty infrastructure is a problem affecting water insecurity in both HICs and LMICs [115], however, improvements are time-intensive and financially costly, and may therefore be unaffordable for poorer communities. After construction, mismatch between who funds and oversees facility operations or excess small facilities can introduce financial complications [116,117]. Furthermore, projected increases in water contamination secondary to climate change will likely increase costs associated with treating water [118]. This could deepen entrenched disparities, as LMICs are expected to experience the greatest increases in water insecurity due to climate change, particularly in the Middle East and North Africa, where underserved communities may be particularly vulnerable [119,120]. Improving the safety and confidence in NPW is crucial in LMICs, as a reliance on alternative beverages can create significant financial burdens for some households. The aggressive marketing and low prices of SSBs in some countries may push households towards selecting less nutritious alternative beverages to reduce costs. Additionally, insufficiently regulated packaged water may pose equal or greater health concerns than local NPW. Working with communities to enhance modular, adaptive, and decentralized water systems can improve water security while working towards the long-term goal of piped water systems [121].

In addition to improving infrastructure and water quality in areas experiencing water insecurity, steps should also be taken to assure the public of water safety. Governmental testing of public water is often not communicated directly to community members [122,123], but finding ways to effectively communicate the safety of NPW through transparent, easily accessible platforms could help reduce selection of alternative beverages. Mistrust of governmental information by marginalized groups may necessitate point-of-use tests or third-party testing and dissemination of results to increase confidence in NPW safety [115,124,125].

Finally, any future interventions to improve water insecurity and decrease selection of alternative beverages must meet population-specific concerns. For example, treating water with chlorine may not be accepted by all communities [115,126]. A focus group with Canadian subarctic Indigenous communities revealed that chlorine was considered a contaminant alongside heavy metals, leading participants to preferentially use water from the land rather than treated tap water. Furthermore, interventions should consider how cultural or community norms may influence alternative beverage selection, especially given that some beverages’ are embedded with traditions, status, and hospitality norms [127–129]. Working closely with community partners and other key local organizations is important for ensuring that feasible and acceptable interventions are promoted [130].

When water insecurity is acutely unavoidable or in the process of improvement, interventions can focus on decreasing the negative health impacts of alternative beverages. For example, organizations or governmental subsidies can provide filters or similar technologies at the community or household level to allow for safe NPW consumption. At the policy level, SSB taxes can shift alternative beverage selection from SSBs to more nutritious alternatives such as packaged water. Furthermore, reducing SSB production would decrease the fresh-water demand in certain water-stressed areas [131]. Finally, single-use plastic taxes or regulations can encourage reformulation of beverage packaging to utilize less or recycled plastics in order to limit the harmful environmental impacts of bottled beverages [132].

The strengths of this scoping review include the collaboration with a health systems librarian to develop the search plan, the comprehensive definition of water insecurity that includes various domains, and the multidisciplinary team involved in developing the conceptual diagram and in determining the scope of the review. One limitation of the study is the broad nature of scoping reviews that prevents aggregating quantitative findings from the included studies. Future research would benefit from using standardized tools such as the Water Insecurity Experiences Scales to better compare individual and household water insecurity across time and settings [133,134]. Another limitation is that, as with most other scoping reviews, a quality assessment step was not included when reviewing the studies. Additionally, this scoping review did not include grey literature which may have included reports from organizations studying water insecurity. Furthermore, it may have missed peer review studies that were not in the searched databases or written in the included languages. This scoping review provided a comprehensive overview of the current literature on the relationship between water insecurity and alternative beverage selection, laying the groundwork for future studies and reviews to fill in the remaining knowledge gaps systematically.

## 5. Conclusion

Climate change is expected to worsen water insecurity through decreasing freshwater availability and increasing hydration needs, exacerbating nutritional concerns, particularly if consumers replace NPW with SSBs. This review highlighted the characteristics of NPW that promote or impede selection, and how that alters the selection of alternative beverages such as packaged water and SSBs. Increases in packaged water or sugar sweetened beverage selection were found by most studies investigating each relationship. Variations in this relationship were seen by key sociodemographic characteristics, however the direction differed by study setting.

## Supplementary Material

Supplementary Material

S1_Scoping Review Checklist

Supporting information

S1 Checklist. Preferred Reporting Items for Systematic reviews and Meta-Analyses extension for Scoping Reviews (PRISMA-ScR) Checklist.

(PDF)

S1 Appendix. Search terms, study characteristics, and extracted details from a scoping review on the role of water insecurity in influencing water and sugar-sweetened beverage choices.

(DOCX)

## Figures and Tables

**Table 1.** Inclusion and exclusion criteria for a scoping review on water insecurity and alternative beverage choices.

Category	Include	Exclude
Population	Human studies of any age. Non-institutionalized population.	Animal studies. Institutionalized population.
Intervention/Exposure	Interventions that alter any domain of water insecurity (availability, accessibility, acceptability and sufficiency for use, or stability across time). Water insecurity (physical or experienced insufficient availability, accessibility, acceptability or sufficiency for use, or stability across time).	Interventions focusing on increasing water selection without altering water insecurity (e.g., behavior change interventions). Water or fluid selection without any mention of insecurity. Agriculturally focused water insecurity.
Outcomes	Studies measuring the selection of beverages other than non-packaged water, such as SSBs, soda, juice, other sweetened beverages, or packaged water (“alternative beverages”). Studies measuring purchasing behavior of alternative beverages. Studies measuring the availability of alternative beverages compared to non-packaged water. Studies describing motivations for purchasing behaviors in water-stressed areas.	Studies measuring overall fluid selection without fluid type sub-categories. Studies that do not directly measure alternative beverage selection or perceptions (e.g., studies theorizing reduced selection due to measured increases in water selection). Studies only examining alcohol selection.
Timing	Studies from all years	N/A
Setting	Any country	N/A
Study Design	Quantitative, qualitative, or mixed methods	Editorials, reviews, grey literature
Language	Studies available in English, Spanish, or Portuguese	Studies not available in English, Spanish, or Portuguese

**Table 2.** Characteristics of studies included in a coping review of water insecurity and alternative beverage choices (n=82).

Characteristic	Number studies reporting
**Publication Year**	2016 (4.8)
Mean (standard deviation)	Range: 1999–2023
**Geographical Region** *	
*North America*	54 (66%)
*Central America*	1 (1%)
*South America*	7 (9%)
*Europe*	10 (12%)
*Asia*	7 (9%)
*Africa*	3 (4%)
*Oceania*	4 (5%)
**World Bank Income Classification (Fiscal Year 2024)** *	
*Lower-middle Income*	6 (7%)
*Upper-middle Income*	20 (24%)
*High Income*	60 (73%)
**Study Design**	
*Intervention*	
*Randomized controlled trial (RCT)*	8 (10%)
*Quasi-experimental*^†^	4 (5%)
*Pilot study*	3 (4%)
*Observational*	
*Case-control*	2 (2%)
*Cross-sectional*	37 (45%)
*Case study*	1 (1%)
*Longitudinal*	5 (6%)
*Qualitative*	15 (18%)
*Mixed methods*	7 (9%)
**Sociodemographic Characteristic Surveyed**	
*Age*	49 (60%)
*Gender*	49 (60%)
*Education*	34 (41%)
*Income*	31 (38%)
*Race/Ethnicity*	28 (34%)

* Some studies include countries across multiple regions.

† As defined by original study authors.

**Table 3.** Number of Studies Showing Variation by Demographic Characteristics in Alternative Beverage Behaviors in Settings of Water Insecurity.

Association with Alternative Beverage Behaviors	Women vs Men n = 22		Higher Income* n = 22		Higher Education n = 16		Older Age n = 23		Race/Ethnicity^‡^ n = 17	
Beverage Type^¶^	PW n = 18	SSB n = 4	PW n = 19	SSB n = 3	PW n = 14	SSB n = 2	PW n = 18	SSB n = 5^†^	PW n = 13	SSB n = 6
**Higher**	7	1	11	0	4	0	1	1	11	4
**Null**	8	0	3	2	5	2	8	0	2	2
**Lower**	3	3	5	1	5	0	9	3	0	0

* Household or Individual income.

† Cells in column do not add to 5 due to one study reporting higher selection among adolescents compared to either older or younger age groups.

‡ Compared to study defined referent group; see S1 Appendix for additional details on index and reference groups.

¶ Packaged water (PW), Sugar-sweetened beverage (SSB).

## Data Availability

All data is referenced throughout the manuscript and listed in the Supporting information.
