# Supplementary material for: The role of water insecurity in influencing water and sugar-sweetened beverage choices: A scoping review
Source: PLOS Sustain Transform. Author manuscript; Available in PMC 2026 Jan 9. (PMC12781548; doi:10.1371/journal.pstr.0000174)
Supplement: Supplementary Material [file NIHMS2122427-supplement-Supplementary_Material.docx]

[Search Terms 3](#_Toc184655969)

[Pubmed 3](#_Toc184655970)

[Scopus 3](#_Toc184655971)

[Global Health 4](#_Toc184655972)

[Embase 4](#_Toc184655973)

[Study Characteristics 6](#_Toc184655974)

[Extracted Details about Non-Packaged Characteristics Relationship with Alternative Beverages 30](#_Toc184655975)

[Perceived Safety 30](#_Toc184655976)

[Packaged Water 30](#_Toc184655977)

[SSBs 31](#_Toc184655978)

[Taste 32](#_Toc184655979)

[Packaged Water 32](#_Toc184655980)

[SSBs 33](#_Toc184655981)

[Convenience/Accessibility 33](#_Toc184655982)

[Packaged Water 33](#_Toc184655983)

[SSBs 34](#_Toc184655984)

[Treatment/Filtering 36](#_Toc184655985)

[Packaged Water 36](#_Toc184655986)

[SSBs 36](#_Toc184655987)

[Cost 37](#_Toc184655988)

[Packaged Water 37](#_Toc184655989)

[SSBs 38](#_Toc184655990)

[Appearance/Turbidity 38](#_Toc184655991)

[Packaged Water 38](#_Toc184655992)

[SSBs 38](#_Toc184655993)

[Temperature 38](#_Toc184655994)

[Packaged Water 38](#_Toc184655995)

[SSBs 38](#_Toc184655996)

[Water Testing 39](#_Toc184655997)

[Packaged Water 39](#_Toc184655998)

[SSBs 39](#_Toc184655999)

[Hardness 39](#_Toc184656000)

[Packaged Water 39](#_Toc184656001)

[SSBs 39](#_Toc184656002)

[Race/Ethnicity Reference Groups 40](#_Toc184656003)

[Packaged Water 40](#_Toc184656004)

[SSB 41](#_Toc184656005)

# Search Terms

## Pubmed

**1841 Resulting Studies**

(beverage[tiab] OR soda[tiab] OR "carbonated beverage"[tiab] OR "carbonated beverages"[tiab] OR "fizzy drink"[tiab] OR “fizzy drinks”[tiab] OR pop[tiab] OR "sugar-sweetened beverage"[tiab] OR "sugar-sweetened beverages"[tiab] OR (sugar[tiab] AND beverages[tiab]) OR "sugared beverages"[tiab] OR (sweetened[tiab] AND beverages[tiab]) OR "sweet drinks"[tiab] OR "sweetened drinks"[tiab] OR "sugary drinks"[tiab] OR juice[tiab] OR juices[tiab] OR nectar[tiab] OR nectars[tiab] OR “fruit drink”[tiab] OR "fruit drinks"[tiab] OR “fruit flavored beverage”[tiab] OR "fruit flavored beverages"[tiab] OR "soft drinks"[tiab]

OR "soft drink"[tiab] OR “energy drink”[tiab] OR "energy drinks"[tiab] OR coffee[tiab] OR tea[tiab] OR “sports beverage”[tiab]  OR "sports beverages"[tiab] OR “sports drink”[tiab] OR "sports drinks"[tiab] OR "bottled water"[tiab]) AND ("Water"[Mesh] OR "Water Resources"[Mesh] OR "Water Supply"[Mesh] OR water[tiab] OR h2o[tiab])

AND (Availability[tiab] OR access[tiab] OR supply[tiab] OR intake[tiab] OR security[tiab] OR insecurity[tiab] OR shortage[tiab] OR unavailable[tiab] OR scarcity[tiab])

## Scopus

**2541 Resulting Studies**

( TITLE-ABS ( beverage ) OR TITLE-ABS ( soda ) OR TITLE-ABS ( "carbonated beverage" ) OR TITLE-ABS ( "carbonated beverages" ) OR TITLE-ABS ( "fizzy drink" ) OR TITLE-ABS ( "fizzy drinks" ) OR TITLE-ABS ( pop ) OR TITLE-ABS ( "sugar-sweetened beverage" ) OR TITLE-ABS ( "sugar-sweetened beverages" ) OR ( TITLE-ABS ( sugar ) AND TITLE-ABS ( beverages ) ) OR TITLE-ABS ( "sugared beverages" ) OR ( TITLE-ABS ( sweetened ) AND TITLE-ABS ( beverages ) ) OR TITLE-ABS ( "sweet drinks" ) OR TITLE-ABS ( "sweetened drinks" ) OR TITLE-ABS ( "sugary drinks" ) OR TITLE-ABS ( juice ) OR TITLE-ABS ( juices ) OR TITLE-ABS ( nectar ) OR TITLE-ABS ( nectars ) OR TITLE-ABS ( "fruit drink" ) OR TITLE-ABS ( "fruit drinks" ) OR TITLE-ABS ( "fruit flavored beverage" ) OR TITLE-ABS ( "fruit flavored beverages" ) OR TITLE-ABS ( "soft drinks" ) OR TITLE-ABS ( "soft drink" ) OR TITLE-ABS ( "energy drink" ) OR TITLE-ABS ( "energy drinks" ) OR TITLE-ABS ( coffee ) OR TITLE-ABS ( tea ) OR TITLE-ABS ( "sports beverage" ) OR TITLE-ABS ( "sports beverages" ) OR TITLE-ABS ( "sports drink" ) OR TITLE-ABS ( "sports drinks" ) OR TITLE-ABS ( "bottled water" ) ) AND ( INDEXTERMS ( water ) OR INDEXTERMS ( "Water Resources" ) OR INDEXTERMS ( "Water Supply" ) OR TITLE-ABS ( water ) OR TITLE-ABS ( h2o ) ) AND ( TITLE-ABS ( availability ) OR TITLE-ABS ( access ) OR TITLE-ABS ( supply ) OR TITLE-ABS ( intake ) OR TITLE-ABS ( security ) OR TITLE-ABS ( insecurity ) OR TITLE-ABS ( shortage ) OR TITLE-ABS ( unavailable ) OR TITLE-ABS ( scarcity ) ) AND ( LIMIT-TO ( LANGUAGE , "English" ) OR LIMIT-TO ( LANGUAGE , "Spanish" ) OR LIMIT-TO ( LANGUAGE , "Portuguese" ) ) AND ( LIMIT-TO ( EXACTKEYWORD , "Human" ) )

## Global Health

**1861 Resulting Studies**

((TI beverage OR AB beverage) OR (TI soda OR AB soda) OR (TI "carbonated beverage" OR AB "carbonated beverage") OR (TI "carbonated beverages" OR AB "carbonated beverages") OR (TI "fizzy drink" OR AB "fizzy drink") OR (TI "fizzy drinks" OR AB "fizzy drinks") OR (TI pop OR AB pop) OR (TI "sugar-sweetened beverage" OR AB "sugar-sweetened beverage") OR (TI "sugar-sweetened beverages" OR AB "sugar-sweetened beverages") OR ((TI sugar OR AB sugar) AND (TI beverages OR AB beverages)) OR (TI "sugared beverages" OR AB "sugared beverages") OR ((TI sweetened OR AB sweetened) AND (TI beverages OR AB beverages)) OR (TI "sweet drinks" OR AB "sweet drinks") OR (TI "sweetened drinks" OR AB "sweetened drinks") OR (TI "sugary drinks" OR AB "sugary drinks") OR (TI juice OR AB juice) OR (TI juices OR AB juices) OR (TI nectar OR AB nectar) OR (TI nectars OR AB nectars) OR (TI "fruit drink" OR AB "fruit drink") OR (TI "fruit drinks" OR AB "fruit drinks") OR (TI "fruit flavored beverage" OR AB "fruit flavored beverage") OR (TI "fruit flavored beverages" OR AB "fruit flavored beverages") OR (TI "soft drinks" OR AB "soft drinks") OR (TI "soft drink" OR AB "soft drink") OR (TI "energy drink" OR AB "energy drink") OR (TI "energy drinks" OR AB "energy drinks") OR (TI coffee OR AB coffee) OR (TI tea OR AB tea) OR (TI "sports beverage" OR AB "sports beverage") OR (TI "sports beverages" OR AB "sports beverages") OR (TI "sports drink" OR AB "sports drink") OR (TI "sports drinks" OR AB "sports drinks") OR (TI "bottled water" OR AB "bottled water"))

AND ((MH Water+) OR (MH "Water Resources+") OR (MH "Water Supply+") OR (TI water OR AB water) OR (TI h2o OR AB h2o))

AND ((TI Availability OR AB Availability) OR (TI access OR AB access) OR (TI supply OR AB supply) OR (TI intake OR AB intake) OR (TI security OR AB security) OR (TI insecurity OR AB insecurity) OR (TI shortage OR AB shortage) OR (TI unavailable OR AB unavailable) OR (TI scarcity OR AB scarcity))

Narrow by Language: - Portuguese, Spanish, English

Narrow by Subject: - man

## Embase

**129 Resulting Studies**

(beverage:ti,ab OR soda:ti,ab OR 'carbonated beverage':ti,ab OR 'carbonated beverages':ti,ab OR 'fizzy drink':ti,ab OR 'fizzy drinks':ti,ab OR pop:ti,ab OR 'sugar-sweetened beverage':ti,ab OR 'sugar-sweetened beverages':ti,ab OR (sugar:ti,ab AND beverages:ti,ab) OR 'sugared beverages':ti,ab OR (sweetened:ti,ab AND beverages:ti,ab) OR 'sweet drinks':ti,ab OR 'sweetened drinks':ti,ab OR 'sugary drinks':ti,ab OR juice:ti,ab OR juices:ti,ab OR nectar:ti,ab OR nectars:ti,ab OR 'fruit drink':ti,ab OR 'fruit drinks':ti,ab OR 'fruit flavored beverage':ti,ab OR 'fruit flavored beverages':ti,ab OR 'soft drinks':ti,ab OR 'soft drink':ti,ab OR 'energy drink':ti,ab OR 'energy drinks':ti,ab OR coffee:ti,ab OR tea:ti,ab OR 'sports beverage':ti,ab OR 'sports beverages':ti,ab OR 'sports drink':ti,ab OR 'sports drinks':ti,ab OR 'bottled water':ti,ab)

AND (Water/exp OR 'Water Resources'/exp OR 'Water Supply'/exp OR water:ti,ab OR h2o:ti,ab)

AND (Availability:ti,ab OR access:ti,ab OR supply:ti,ab OR intake:ti,ab OR security:ti,ab OR insecurity:ti,ab OR shortage:ti,ab OR unavailable:ti,ab OR scarcity:ti,ab)

AND “human”/de

# Study Characteristics

| **Study Year** | **Title** | **Author** | | | **Country/**  **Region** | **Aim of study** | **Study design** | | **Population description** | **Sample Size** | **Intervention/**  **Exposure Description** | |
| --- | --- | --- | --- | --- | --- | --- | --- | --- | --- | --- | --- | --- |
| 2018 | Does the Mexican sugar-sweetened beverage tax have a signaling effect? ENSANUT 2016 | Alvarez-Sanchez et al. | | | Mexico | To explore whether awareness of the SSB tax and opinion about its potential to reduce SSB intake, as well as psychosocial and environmental determinants of SSB consumption, are associated with current consumption of taxed SSBs, and with self-reported changes in consumption of SSBs since the SSB tax was passed | Cross sectional study | | Mexican adults | 6650 | Availability of free/low-cost potable water | |
| 2007 | A Family-based Intervention to Promote Healthy Lifestyles in an Aboriginal Community in Canada | Anand et al. | | | Canada | To determine if a household-based lifestyle intervention is effective at reducing energy intake and increasing energy expenditure to inform the development of a large-scale household-based intervention in an Aboriginal com- munity | Randomized controlled trial | | Aboriginal households from Six Nations Reserve | 57 households, 174 individuals | Household-based lifestyle intervention + provision of filtered water | |
| 2021 | Tendencies towards bottled drinking water consumption: Challenges ahead of polyethylene terephthalate (PET) waste management | Aslani et al. | | | Iran | To examine the logic behind the tendencies towards bottled drinking water usage, while potable drinking water with a standard quality is available. And to estimate the amount of waste generated from these bottles and present managing principles to resolve the issue | Cross sectional study | | Adult bottled water users | 120 | Perceived tap water concerns (authors stated that water quality is high in studied city) | |
| 2012 | Consumption Patterns and Perception on Intake of Drinking Water in Klang Valley, Malaysia | Azlan et al. | | | Malaysia | To investigate the consumption and perception of consumers related to tap and commercial drinking water available in Malaysia (Klang Valley areas) | Cross sectional study | | Adults | 225 | Perception on usage of drinking water (quality and price) | |
| 2017 | Formative Research to Design a Promotional Campaign to Increase Drinking Water among Central American Latino Youth in an Urban Area | Barrett et al. | | | USA | To understand beverage choice motivation, behaviors, and preferences, and test concepts and messages in order to design promotional materials for drinking water that were appealing to Latino youth who live in the DC urban metropolitan area | Qualitative research | | Latino youth (6-18 y/o) | 61 | Beverage consumption environments | |
| 2022 | Perceptions of tap water associated with low-income Michigan mothersâ€™ and young childrenâ€™s beverage intake | Bauer et al. | | | USA (Michigan) | To quantify perceptions of home tap water and tap water in general among low-income Michigan mothers and examine associations between tap water perceptions and beverage intake among mothers and their young children (aged 0 through 4 years) | Cross sectional study | | Medicaid-insured mothers with children aged 0-4 residing in Michigan but outside of Flint | 500 mothers | Perceptions of tap water | |
| 2013 | Parental and Home Environmental Facilitators of Sugar-Sweetened Beverage Consumption Among Overweight and Obese Latino Youth | Bogart et al. | | | USA, California | To explore parental and home environmental facilitators of sugar-sweetened beverage (SSB) and water consumption among obese/overweight Latino youth | Qualitative research | | Latino youth (10-18 y/o) that consume SSB and parents | 55 parents/55 children | Beliefs about water | |
| 2020 | Tapping Out: Influence of Organoleptic and Perceived Health Risks on Bottled Versus Municipal Tap Water Consumption Among Obese, Low Socioeconomic Status Pediatric Patients | Collier et al. | | | USA, North Carolina | To assess the water consumption habits of obese treatment seeking patients from this high-risk region to determine what percentage might be at risk of exposure to obesogenic PPCPs from drinking  municipal (largely surface) water versus well water | Cohort study | | Families seeking treatment at a NC clinic for an obese child aged 6 months to 10 years | 267 families | Domestic water source | |
| 2015 | Assessing human exposure to inorganic arsenic in  high-arsenic areas of Latium: a biomonitoring study  integrated with indicators of dietary intake | Cubadda et al. | | | Italy | To assess inorganic arsenic exposure and metabolism in residents of the largest arsenic-affected area in Italy | Cross sectional study | | Children and adults living in Latium | 269 | Inorganic arsenic in water and food | |
| 2018 | Water intake among Ghanaian youth aged 15-34 years: quantitative and qualitative evidence | Doegah & Amoateng | | | Ghana | To examine the association between selected socio-demographic factors and the consumption of potable water among Ghanaian youth aged 15-34 years. | Other: Quantitative and cross-sectional | | Ghana youth (15-34 years) | 8 focus groups | Perception regarding water consumption | |
| 2013 | Water and beverage consumption among adults in the United States: cross-sectional study using data from NHANES 2005-2010 | Drewnowski et al. | | | USA | To evaluate the consumption of plain water (tap and bottled) and total water among US adults by age group (20-50y, 51-70y, and ≥71y), gender, income-to-poverty ratio, and race/ethnicity | Cross sectional study | | US adults (>20 y/o) | 15702 | Beverage selection | |
| 2010 | Differences in water consumption choices in Canada: the role of socio-demographics, experiences, and perceptions of health risks | Dupont et al. | | | Canada | To investigate the factors that influence these choices and whether choosing to either filter or purchase water is linked to perceptions of health concerns with respect to tap water. | Cross sectional study | | Canadian adult internet users | 1633 | Tap water perceptions | |
| 2014 | Drinking Water Management: Health Risk Perceptions and Choices in First Nations and Non-First Nations Communities in Canada | Dupont et al. | | | Canada | To investigate differences in beliefs about health risks from tap water and bottled water purchases of residents in four First Nations communities and non-First Nations Canadians across Canada. The second objective was to determine whether observed differences in health concerns translate into health risk avoidance expenditures and  response | Other: Mixed methods (cross-sectional & qualitative) | | First Nations and diverse non-First nations Canadians | Survey A: 301  Survey B: 86  Survey C: 1307 | Perception of Water Sources | |
| 2016 | Beverage consumption in an Alaska Native village: a mixed-methods study of behavior, attitudes and access | Elwan et al. | | | USA | To assess the frequency of SSB, water and milk consumption; to ascertain the attitudes towards consumption of water, milk and SSB of residents of a rural, Interior Alaska Native (Athasbascan) community; and to assess rural access to water, milk and SSB | Other: Mixed methods | | Alaska Native children and adults residing in rural Alaska | 2 focus groups (21 people), 7 early head start program instructors, 69 beverage FFQ respondents | Beverage behavior, attitudes, and access | |
| 2013 | Knowledge about simple water consumption in adults of low socioeconomic status from the city of Cuernavaca, Mexico | Espinosa-Montero et al. | | | Mexico | To describe everyday knowledge about plain water and its consumption in adults of low socioeconomic status, residing in the city of Cuernavaca, Morelos, Mexico | Qualitative research | | Low SES adults living in Cuernavaca | 49 | Knowledge about plain water | |
| 2023 | Water Security Experiences and Water Intake Among Elementary Students at Low-Income Schools: A Cross-Sectional Study | Ezennia et al. | | | USA | To examine how students’ experiences of water security at school relate to their intake of water in the setting | Cross sectional study | | Elementary school students (low-income) | 651 | School drinking water security experiences | |
| 2019 | Supporting healthy drink choices in remote Aboriginal and Torres Strait Islander communities: a community-led supportive environment approach | Fehring et al. | | | Australia | To assess change in community readiness,  awareness of social marketing messaging,  drink availability, and consumption of water  and sugary drinks in remote Aboriginal and  Torres Strait Islander communities | Other: Qualitative and community intervention | | Aboriginal and Torres Strait Islander community members | 3 communities (97 participants interviewed) | Infrastructure changes to improve availability and access, such as installing chilled water bubblers, relaying drink fridges in stores to promote bottled  water and displaying water promotion signage in community settings | |
| 2014 | A Community Engagement Process Identifies Environmental Priorities to Prevent Early Childhood Obesity: The Children’s Healthy Living (CHL) Program for Remote Underserved Populations in the US Affiliated Pacific Islands, Hawaii and Alaska | Fialkowski et al. | | | USA, (Pacific Islands, Hawaii, Alaska) | To describe the community engagement process (CEP) used by the Children’s Healthy Living (CHL) Program for remote underserved minority populations in the USAPI/HI/AK. To highlight the overall priorities for environmental intervention strategies identified by communities for community-based, environmentally focused childhood obesity prevention in the USAPI/HI/AK Region and the lessons learned from the CEP. | Qualitative research | | Remote Underserved communities in the USAPI, Hawaii, and Alaska | 20 communities | Improved drinking water access/facilities (primarily school and public places): identified priority by American Samoa, CNMI, Guam, Hawaii | |
| 2015 | What do Indian children drink when they do not receive water? Statistical analysis of water and alternative beverage consumption from the 2005â€“2006 Indian National Family Health Survey | Fledderjohann et al. | | | India | To examine the prevalence of children who receive no water in India and to investigate what children drink when they are reported to have no water | Cross sectional study | | Mother's reporting intake of children aged 6-59 months | 30,656 children; 22,668 mothers | Receipt of water | |
| 2020 | Evaluation of bottled water quality by determining nitrate concentration | | Fortunato et al. | | Argentina, Buenos Aires | To evaluate the quality of different commercial bottled waters from different locations by determining nitrate concentration, (b) to relate bottled water quality with water access in the different locations, (c) to analyze public awareness about bottled water quality and consumption habits of the population in the urban area of Buenos Aires | Cross sectional study | | Shop owners and Argentinian adults in C.A.B.A(high water access) or Malvinas (low water access) | 364 | Water perceptions and preferences | |
| 2016 | Trust matters: Why augmenting water supplies via desalination may not overcome perceptual water scarcity | | | Fragkou et al. | Latin America (Mexico and Chile) | To provide empirical evidence which challenges the assumption that a state-of-the-art technical solution to water provision will address water quality (and water scarcity) concerns. | | Other: Case study | Households in Los Cabos, Mexico and Antofagasta, Chile | 155 households (Mexico), 100 households (Chile) | | Installation of desalination plants |
| 2020 | PET-Bottled Water Consumption in View of a Circular Economy: The Case Study of Salento (South Italy) | | | Gambino et al. | Italy | To investigate the consumption of drinking water by the population living in the Province of Lecce with particular reference to the main supplies used and the factors related to the choice of supply including the consumers’ perception about the quality and safety of the water from public system. To measure the consumption of PET-bottled water. | | Qualitative research | Adults and children living in Province of Leece | 4137 | | Residence in Leece (known for increasingly contaminated ground water) |
| 2020 | Bottle or tap? Toward an integrated approach to water type consumption | | | Geerts et al. | Belgium | To analyze the main reasons why people consume tap and bottled water in Flanders, Belgium | | Cross sectional study | Flemish adults | 2309 | | Tap water perceptions |
| 2018 | Consumer water quality evaluation of private and public drinking water sources | | | Gholson et al. | USA (Texas) | To examine differences in the perception of water quality based on an individual’s primary drinking water source. | | Cross sectional study | Texan adults | 491 | | Perception of home drinking water safety |
| 2017 | The school environment and sugar-sweetened beverage consumption among Guatemalan adolescents | | | Godin et al. | Guatemala | To identify individual-level characteristics associated with SSB consumption and describe school characteristics that may influence students’ SSB intake | | Cross sectional study | Guatemala secondary school students | 1042 | | School beverage environment |
| 2019 | Effects of a multipronged beverage intervention on young children’s beverage intake and weight: a cluster-randomized pilot study | | | Grummon et al. | USA (northern California) | To evaluate the pilot intervention’s impact on children’s weight (overweight/obese status (primary outcome), BMI and BMI percentile) and beverage consumption, with a goal of determining the promise of the intervention and providing the data necessary to calculate sample size needs for larger trials | | Other: Pilot study | Childcare centers with children aged 2-5 years | 4 childcare centers (7 classrooms, 164 children(154 included in final analysis)) | | Environmental change component: increasing children’s access to water at childcare centers and at home; Research team tested classroom water sources for lead & remediated as needed. Classrooms received personalized water bottles for each child to use at child care. Classrooms received child-friendly water pitchers and cups for serving water with meals and snack. Parents received personalized water bottles for child to use at home |
| 2016 | How do people make a decision on bottled or tap water? Preference elicitation with nonparametric bootstrap simulations | | | Gungor-Demirci et al. | USA | To elicit consumer preferences toward drinking water and the associated attributes and choices via a survey | | Cross sectional study | Civil and Environmental Engineering students | 141 | | Water selection preference |
| 2015 | Impact of a Water Intervention on Sugar-Sweetened Beverage Intake Substitution by Water: A Clinical Trial in Overweight and Obese Mexican Women | | | Hernandez-Cordero & Popkin | Mexico | To describe challenges to promote and achieve an  increase in water intake and present key findings from a clinical trial examining the effects of substituting water for SSB on triglyceride levels, weight and other cardiometabolic factors in overweight/obese Mexican women | | Randomized controlled trial | Mexican women with BMI 25-39 | 240 | | Provision of education or water+education |
| 2019 | Perceptions of water and sugar-sweetened beverage consumption habits among teens, parents and teachers in the rural south-western USA | | | Hess et al. | USA, rural New Mexico | To gain a more thorough understanding of the perceptions surrounding the safety and appeal of drinking water, SSB preferences and consumption habits, and reasons behind those consumption habits, as well as factors specific to the rural context that might influence beverage choice among youths. To explore perceptions of both water and SSB, and thus contributes to our understanding of the interrelated factors which can guide efforts to decrease SSB and increase water consumption. | | Qualitative research | Middle- and High-school students | 40 total; 1 MS focus group(FG), 1 HS FG, 1 parent FG, 1 teacher FG | | Beverage consumption habits |
| 2016 | Worksite nutrition supports and sugar-sweetened beverage consumption | | | Hipp et al. | USA, Missouri | To examine access and use of twelve unique worksite appliances, cafeteria, services, and supports for nutrition behaviors and their association with self-reported consumption across five different SSB categories. | | Cross sectional study | Employed adults in Missouri | 2015 | | Perceived access to nutrition behavior supporting workplace features |
| 2014 | A qualitative study of the factors that influence mothers when choosing drinks for their young children | | | Hoare et al. | Australia, Victoria’s Barwon South Western Region | To investigate drivers of beverage consumption for young children | | Qualitative research | Mothers of infants (6-12 months) | 32 | | Attitudes and perceptions towards drink choice, water quality and oral health. |
| 2020 | Drinking water improvements and rates of urinary and  gastrointestinal infections in Galapagos, Ecuador: assessing household and community factors | | | Houck et al. | Ecuador, Galapagos | To contextualize the link between contaminated water, exposures and infections | | Cross sectional study | Mothers of children 2-10 y/o in urban area of San Cristobal | 121 mothers, 168 children | | New drinking water treatment plant installation |
| 2011 | Bottled Water: United States Consumers and Their Perceptions of Water Quality | | | Hu et al. | USA | To identify explanatory factors for bottled water consumption | | Cross sectional study | US adults | 5823 | | Water perceptions |
| 2020 | Promoting healthy beverage consumption habits among elementary school children: results of the Healthy Kids Community Challenge “Water Does Wonders” interventions in London, Ontario | | | Irwin et al. | Canada, Ontario | To evaluate the effectiveness of a series of school-based interventions that combined water and nutrition education with environmental changes to support water consumption on increasing children’s water consumption and decreasing their SSB consumption | | Randomized controlled trial | 4-8 grade children (~8-14 y/o) in neighborhoods with high community needs | 17 elementary schools; 931 parent-child dyads | | New automatic water bottle filling station in schools (all) and education (2 arms) |
| 2021 | Water consumption, sugary drinks and use of drinking fountains in secondary schools of the Program National School Drinking Fountains Mexico City | | | Jimenez-Aguilar et al. | Mexico | To describe beverages consumption practices,  and school drinking fountains utilization among secondary school’s students from National School Drinking Fountains Program | | Other: Qualitative and Cross-Sectional | Secondary school students, public schools | 617 | | Drinking fountain utilization and plain water consumption barriers/facilitators |
| 2012 | Determinants of bottled and purified water consumption: results based on an OECD survey | | | Johnstone and Serret | Australia, Canada, Czech Republic, France, Italy, Korea, Mexico, the Netherlands, Norway and Sweden | To analyze the determinants of a household’s decision to purchase bottled water or invest in a purification system | | Cross sectional study | Adults residing in OCED countries | 10,000 households | | Determinants of bottled water consumption |
| 2006 | Public perceptions of drinking water: a postal survey of residents with private water supplies | | | Jones et al. | Canada | To investigate the public perceptions of water from private water supplies in the City of Hamilton, Ontario (Canada), with the intent of informing public education and outreach strategies within that population. | | Cross sectional study | Hamilton (Ontario) resident with private water supplies | 246 | | Private water supply |
| 2022 | Water Wins, Communication Matters: School-Based Intervention to Reduce Intake of Sugar-Sweetened Beverages and Increase Intake of Water | | | Kamin et al. | Slovenia | To develop and test interventions that educate, enable, and engage primary school children their parents and relatives, and teachers and other school co-workers to reduce SSB intake and increase water consumption | | Other: Quasi-experimental | Slovenian primary schools | 4 primary schools, 672 children (10-16 y/o) | | Offering water/non-sugary beverages in schools instead of SSB + promotional material |
| 2019 | Providing Students with Adequate School Drinking Water Access in an Era of Aging Infrastructure: A Mixed Methods Investigation | | | Kenney et al. | USA | To develop a comprehensive understanding of the impact of different systems for delivering water to students | | Other: Mixed methods (qualitative and cross-sectional) | Urban US schools | 6 schools | | Quality and availability of drinking water + perceptions of water availability |
| 2017 | The switch to refillable bottled water in Indonesia: a serious health risk | | | Komarulzaman et al. | Indonesia | To investigate the motives for households’ decision to switch from piped water to refillable bottled water as their main source of drinking water | | Other: Longitudinal | Indonesian households | 63276 households | | Switch to refillable bottled water |
| 1999 | Avoiding health risks from drinking water in Moscow: An empirical analysis | | | Larson and Gnedenko | Russia | To analyze the types and degree of avoidance measures that are used by households in Moscow | | Cross sectional study | Households in Moscow | 615 households | | Opinions of water quality |
| 2021 | Drinking Water Consumption Patterns among Private Well Users in Ontario: Implications for Exposure Assessment of Waterborne Infection | | | Lavallee et al. | Canada, Ontario | To examine the consumption patterns of private well users in Ontario while considering potentially influential underlying sociodemographics, household characteristics, and experiential factor | | Cross sectional study | Private well using adults living in Ontario | 1138 | | Household well water |
| 2019 | A randomized trial of a multi-level intervention to increase water access and appeal in community recreation centers | | | Lawman et al. | USA, Philadelphia | To determine the effectiveness of the “Hydrate Philly” multi-level intervention to increase water access and appeal in community recreation centers in increasing center-level water intake as measured by water flow meters, and decreasing the purchase of outside beverages as measured by observations of youth visiting recreation centers | | Randomized controlled trial | Philadelphia community centers in low-income neighborhoods | 29 community centers | | Water testing and hydration station installation |
| 2020 | Hydrate Philly: An Intervention to Increase Water Access and Appeal in Recreation Centers | | | Lawman et al. | USA, Philadelphia | To test the effectiveness of an intervention to in- crease water use in recreation centers by improving water access and appeal through both built environment and sociocultural strategies in urban, low-income, and racially/ethnically diverse communities | | Randomized controlled trial | Philadelphia recreation centers in low-income neighborhoods | 28 recreation centers | | Installation of water fountains with bottle fillers and testing/communication of results for water |
| 2020 | Household coping strategies associated with unreliable water supplies and diarrhea in Ecuador, an upper-middle-income country | | | Lee et al. | Ecuador | To examine how access to an unreliable water supply relates to household behaviors around water use, and to assess the association between access to an unreliable system, household water use behaviors, and diarrheal disease | | Other: Longitudinal | Households in Borbon Ecuador | 342 initial, 202 f/u | | Unreliable/Contaminated drinking water; damaged water treatment plant in 2009; decreasing reliability over time (in the latter half of 2009, households had access to piped water 79% of the time, compared to 63% by 2017) |
| 2023 | Don’t Drink the Water! The Impact of Harmful Algal Blooms  on Household Averting Expenditure | | | Liu & Klaiber | USA, Toledo, Ohio | To describe averting behaviors after water crisis | | Other: Longitudinal | Toledo Households (Cleveland households as control) | 482 households | | Harmful algal bloom causing water crisis |
| 2005 | Does the provision of cooled filtered water in secondary school cafeterias increase water drinking and decrease the purchase of soft drinks? | | | Loughridge & Barratt | UK, North Tyneside | To measure the effect of health promotion and the free provision of cooled filtered water on the consumption of water and soft drinks | | Other: Pilot study (intervention + focus group) | Low-income secondary schools | 3 schools, 2965 students | | Provision of free cooled filtered water in cafeterias |
| 2011 | FACTORS INFLUENCING THE CONSUMPTION AND STANDARDS OF BOTTLED DRINKING WATER IN NAIROBI-KENYA | | | Mbagaya and Mbato | Kenya | To determine the extent of consumption, brand choice, perceived reasons for consumption, standards and average monthly expenditure on bottled/packaged water among Nairobi residents | | Cross sectional study | Nairobi residents | 120 | | Beverage perceptions |
| 2014 | Risk Factors Associated with the Choice to Drink Bottled Water and Tap Water in Rural Saskatchewan | | | McLeod et al. | Canada, rural Saskatchewan | To investigate risk factors associated with choices to drink bottled water and tap water in rural Saskatchewan | | Cross sectional study | Rural households in Saskatchewan | 2065 households | | Perceptions of quality and health risks from drinking water |
| 2019 | Lack of in-home piped water and reported consumption of sugar-sweetened beverages among adults in rural Alaska | | | Mosites et al. | USA, Alaska | To assess whether a community water service is associated with the frequency of sugar-sweetened beverages (SSB) consumption, obesity, or perceived health status in rural Alaska | | Cross sectional study | Rural Alaskan Adults | 887 | | Community water access |
| 2019 | Quantification of Groundwater Exploitation and Assessment of Water Quality Risk Perception in the Dar Es Salaam Quaternary Aquifer, Tanzania | | | Mussa et al. | Tanzania | To quantify the extent of exploitation of groundwater in the Dar es Salaam aquifer; analyzing water quality risk perception, assessing averting actions to groundwater quality and quantity problems, and establishing levels of groundwater reliability and importance for meeting household water needs | | Cross sectional study | Households in Dar es Salaam using groundwater | 200 households | | Groundwater usage |
| 2023 | Knowing Is Not Doing: A Qualitative Study of Parental Views on Family Beverage Choice | | | Newman et al. | USA, North Carolina | To understand, in a diverse real-world patient population, what parents viewed as the primary drivers of their family’s beverage choices, and explore how these drivers could be addressed in order to make changes to beverage consumption | | Qualitative research | Families of children 1-8 y/o who consume >/=2 sweet drinks/d | 39 | | Parental views on drivers of beverage selection |
| 2012 | The relationship of perceptions of tap water safety with intake of sugar-sweetened beverages and plain water among US adults | | | Onufrak et al. | USA | To examine demographic differences in perceptions of tap water safety and determine if these perceptions are associated with intake of SSB and plain water | | Case control study | US adults | 3787 | | Perceptions of tap water safety |
| 2014 | Perceptions of Tap Water and School Water Fountains and Association With Intake of Plain Water and Sugar-Sweetened Beverages | | | Onufrak et al. | USA | To examine cognitive evaluations of tap-water safety and school water fountains among youth and how these evaluations relate to consumption of plain water and SSB. To assess whether the relationship between tap water and water fountain perceptions with SSB and water intake differs according to race/ethnicity | | Cross sectional study | Households with children aged 9-19 | 1044 | | Tap and school water perceptions |
| 2019 | Perceptions of drinking water safety and their associations with plain water intake among US Hispanic adults | | | Park et al. | USA | To describe sociodemographic differences in perceptions of drinking water safety and to examine whether there are associations between such perceptions and plain water intake among US Hispanic adults | | Cross sectional study | US Hispanic adults | 1000 | | Perception of tap water safety |
| 2023 | Perceptions of Water Safety and Tap Water Taste and Their Associations With Beverage Intake Among U.S. Adults | | | Park et al. | USA | To examine differences in perceptions of tap water (TW) and bottled water (BW) safety and TW taste and their associations with plain water (PW) and sugar-sweetened beverage (SSB) intake | | Case control study | US adults | 4041 | | Tap water perceptions |
| 2019 | Agua4All: Providing Safe Drinking Water in Rural California Communities | | | Patel et al. | USA, California | To examine how bottle-filling stations dispensing safe water (hereinafter, water stations) coupled with either 1)limited promotion (signage, reusable water bottles provided by the study) or 2) limited promotion and the community’s own promotional activities (site-led promotion) affected intake of water in low-income, rural communities with a history of contaminated drinking water | | Other: Quasi-experimental | Rural California communities | 12 sites, 2 communities | | Bottle filling station or bottle filling station+promotional support |
| 2011 | Increasing the Availability and Consumption of Drinking Water in Middle Schools: A Pilot Study | | | Patel et al. | USA, Los Angeles, CA | To examine whether provision of drinking water, coupled with education and promotional activities, was related to increased consumption of water and decreased consumption of SSBs among middle school students in Los Angeles, California | | Other: Quasi-experimental | Low income LA middle school | 1 middle school (7th graders assessed) + 1 control middle school | | Provision of cold, filtered tap water in the school cafeteria; distribution of reusable water bottles to all school staff and students; implementation of school-wide promotional activities; and education regarding the benefits of drinking water |
| 2010 | Perceptions About Availability and Adequacy of Drinking Water in a Large California School District | | | Patel et al. | USA, California | To investigate the availability and adequacy of drinking water in schools; attitudes about, barriers to, and facilitators of providing drinking water in schools; and ideas for increasing water consumption among students | | Qualitative research | California Unified School District middle schools | 26 stakeholders | | School drinking water perception |
| 2019 | Consumer perception of water quality during an off-flavor event in Fortaleza-Brazil | | | Pestana et al. | Brazil, Fortaleza | To determine the influence of the socio-economic background of the volunteers on the perception of off-flavors in water and its affecting factors, maintaining the same educational level and age and to determine perceptions and usage of tap water | | Cross sectional study | Undergraduate students at Fortaleza university | 308 first survey, 374 second survey | | Water perceptions |
| 2016 | Effect and Process Evaluation of a Cluster Randomized Control Trial on Water Intake and Beverage Consumption in Preschoolers from Six European Countries: The ToyBox-Study | | | Pinket et al. | Belgium, Bulgaria, Germany, Greece, Poland, Spain | To examine the effect of the ToyBox-intervention (cluster randomized controlled trial) on water intake and beverage consumption in European preschoolers and to investigate if the intervention effects differed by implementation score of kindergartens and parents/caregivers | | Randomized controlled trial | Preschoolers from 6 European countries | 4964 preschoolers | | Classroom water drinking station implementation and education |
| 2013 | Perceptions of bottled water consumers in three Brazilian municipalities | | | Queiroz et al. | Brazil | To advance the current knowledge of the factors that underlie bottled water consumption in Brazil, including the preference for specific bottled water characteristics and perceptions of tap water. | | Qualitative research | Brazilian adults in predominantly urban areas | 30 | | Amount and value of water consumption |
| 2022 | Use of a Water Filter at Home Reduces Sugary Drink Consumption among Parents and Infants/Toddlers in a Predominantly Hispanic Community: Results from the Water Up!@ Home Intervention Trial | | | Reese et al. | USA, Washington DC | To test the effects of a home-based intervention designed to replace SSBs with tap water and reduce excess juice consumption among parents and their infants/toddlers | | Randomized controlled trial | Parents of infants/toddlers (6mo-3y) participating in Early Heard Start programs | 92 parents | | Provision of a water filter or water filter + education |
| 2015 | Drinking water intake and source patterns within a USA Mexico border population | | | Regnier et al. | USA (Texas) and Mexico (Chihuahua) | To identify water intake and source patterns among a population that resides in a hot, arid region on the USA Mexico border. | | Cross sectional study | Households in the Texas/Mexico border | 579 | | Factors affecting water consumption |
| 2013 | The lack of water and its implications regarding feeding practice in Turbo, Antioquia | | | Rodriguez-Villamil et al. | Colombia | To describing water-related perceptions and practice and how slum dwellers lacking public service coverage in the town of Turbo in the Antioquia Department, Colombia, approach this. | | Qualitative research | Residents living in slums | 18 interviews | | Water availability and water-related perceptions |
| 2023 | Drink Tap: A Multisector Program to Promote Water Access and Intake in San Francisco Parks | | | Rosenthal et al. | USA, California (San Francisco) | To describe how an intervention that combined access to and promotion of public tap water stations with existing SSB taxes, compared with SSB taxes alone, affected beverage intake habits in parks in low-income neighborhoods | | Other: Quasi-experimental |  | 30 parks, 960 park visitor interviewees | | Installation of public tap water stations in parks and public spaces + water promotion; concurrently when SSB city taxes were implemented |
| 2022 | Using Water Intake Dietary Recall Data to Provide a Window into US Water Insecurity | | | Rosinger | USA | To demonstrate how water intake variables from dietary recall data relate to and predict a key water insecurity proxy, that is, tap water avoidance | | Cross sectional study | US adults | 32,329 | | Tap water avoidance |
| 2018 | Disparities in plain, tap and bottled water consumption among US adults: National Health and Nutrition Examination Survey (NHANES) 2007-2014 | | | Rosinger et al. | USA | To determine beverage intake and determinants of intake | | Cross sectional study | US adults (>20 years) | 20,676 | | Water filtration (aim 3) |
| 2022 | How providing a low-cost water filter pitcher led Latino parents to reduce sugar-sweetened beverages and increase their water intake: explanatory qualitative results from the Water Up! @Home intervention trial | | | Santillan-Vazquez et al. | USA, Washington DC | To explain results of the Water Up!@Home randomized controlled trial where low-income parents in both groups had reported statistically significant reductions in sugar-sweetened beverages (SSB) and increases in water intake post-intervention | | Qualitative research | Low-income Latino parents of infants/toddlers who had participated in the Water Up! @Home randomized controlled trial | 32 interviews | | Prior participation in water filter provision RCT |
| 2015 | Water insecurity in Canadian Indigenous communities: some inconvenient truths | | | Sarkar et al. | Canada | To explore the water insecurity, coping strategies and associated health risks in a small and isolated sub-Arctic Indigenous (Inuit) community in Canada | | Other: Mixed (qualitative and quantitative) | Black Tickle-Domino community members | 5 key informant interviews  4 focus groups (43 people total) | | Water insecurity |
| 2010 | Water consumption beliefs and practices in a rural Latino community: implications for fluoridation | | | Scherzer et al. | USA, California | To examine water consumption beliefs and practices among Latino parents of young children in a rural community | | Qualitative research | Latino parents living in a rural community (caregiver of child 1-5y/o) | 46 | | Water perceptions |
| 2022 | The Effect of a Product Placement Intervention on Pupil’s Food and Drink Purchases in Two Secondary Schools: An Exploratory Study | | | Spence et al. | England | To explore the impact of re-positioning sweet-baked goods, fruit, sugar-sweetened beverages (SSBs) and water on pupil’s lunchtime purchases in two secondary schools in North-East England | | Randomized controlled trial | Secondary schools located in "more deprived areas" | 2 secondary schools, 1734 students | | Re-positioning selected food and drinks to increase and decrease accessibility of healthier and less healthy items |
| 2020 | Drinking Water Consumption Patterns: An Exploration of Risk Perception and Governance in Two First Nations Communities | | | Spicer et al. | Canada, Dene Tha’ First Nation and Kátł’odeeche First Nation | To determine how risk perception and other social-economic variables influence individual drinking water consumption patterns (including water from the land and water from home); (ii) explore how political jurisdiction and associated infrastructure and regulations influence individual drinking water consumption patterns | | Qualitative research | Local leaders in the Dene Thaâ€™ First Nation and Kátł’odeeche First Nation | 99 interviews | | Perceptions of drinking water |
| 2020 | Drinking Water Investigation of Hill Tribes: A Case Study in Northern Thailand | | | Sudsandee et al. | Thailand | To investigate the system of drinking water management, and detect its quality, including assessing people’s knowledge and practices relevant to their daily drinking water consumption | | Cross sectional study | Hill tribe members | 2115 persons, 425 households | | Drinking water |
| 2020 | Sugar-sweetened beverage consumption among Indigenous Australian children aged 0-3 years and association with sociodemographic, life circumstances and health factors | | | Thurber et al. | Australia | To explore beverage intake and associations between sugar-sweetened beverage (SSB) intake and sociodemographic, life circumstances, health and well-being factors in a national cohort of Indigenous children | | Cross sectional study | Families of Indigenous children aged 0-3 years, in the Longitudinal Study of Indigenous Children. | 933 families | | sociodemographic, life circumstances, and health and well-being factors |
| 2009 | Willingness to pay for safe drinking water: Evidence from Parral, Mexico | | | Vasquez et al. | Mexico (Chihuahua state, Parral) | To elicit household willingness to pay responses for safe and reliable drinking water in Parral, Mexico | | Cross sectional study | Households in Parral, Mexico | 398 | | Water perceptions and practices |
| 2015 | The Relative Weights of Direct and Indirect Experiences in the Formation of Environmental Risk Beliefs | | | Viscusi and Zeckhauser | USA | To examine the perception of, and responses to, morbidity risks from tap water. | | Cross sectional study | US adults | 1014 | | Prior home tap water illness (direct or indirect) |
| 2010 | Feasibility and Impact of Placing Water Coolers on Sales of Sugar-Sweetened Beverages in Dutch Secondary School Canteens | | | Visscher et al. | Netherlands | To investigate the feasibility and effectiveness of placing water coolers on sugar-sweetened beverage sales at secondary schools  (age 12-18 years) in the city of Zwolle, the Netherlands | | Other: Pilot study | Secondary schools (age 12-18 years) | 6 schools, 5866 students | | Water coolers were placed in the canteen (no additional information/education provided) |
| 2013 | The effect of seasonal climate on bottled water distribution in rural Cambodia | | | White et al. | Cambodia, Battambang province | To assess bottled water as a means of providing rural communities with access to safe drinking water throughout the year. | | Cross sectional study | Rural communities in the Battambang province, Cambodia | 240 households | | Drinking water source |
| 2022 | Before and After the Flint Water Crisis: Changes in the Consumption of Sugary Beverages in Residents Affected by Lead Contamination | | | Wierda et al. | USA, Michigan | To examine the changes in the type and frequency of beverage  consumption of Genesee County, Michigan residents before and after the Flint water crisis (FWC) | | Other: Longitudinal | Adults living in Genesee Count, Michigan | 1825 | | Flint Water Crisis |
| 2020 | Motivators of and Barriers to Drinking Healthy Beverages among a Sample of Diverse Adults in Bronx, NY | | | Wippold et al. | USA (New York) | To identify statistically significant differences in the levels of endorsement of healthy beverage motivators and barriers in association with gender and race/ethnicity | | Cross sectional study | Adults in Bronx, New York | 639 | | Motivators/Barriers to beverage selection |
| 2012 | Qualitative Application of the Theory of Planned Behavior to Understand Beverage Consumption Behaviors among Adult | | | Zoellner et al. | USA, Virginia | To investigate culturally specific attitudes, subjective norms, and perceived behavioral control constructs related to the consumption of SSB, water, and artificially sweetened beverages | | Qualitative research | Adults in rural southwest Virginia who consume 1+ cup of SSB/d | 8 focus groups, 54 participants | | Beverage-specific attitudes, subjective norms, perceived behavioral control, and intentions (including water source) |

# Extracted Details about Non-Packaged Characteristics Relationship with Alternative Beverages

## Perceived Safety

### Packaged Water

| **Study** | **Finding** |
| --- | --- |
| Queiroz 2013 | 100% perceived bottled water as safer than tap. |
| Dupont 2014 | Average 52% perceived bottled water as safer than tap. |
| Fortunato 2020 | 39% perceived bottled water as safer than tap |
| Jones 2006 | 68% perceived bottled water as safer |
| Onufrak 2012 | 26% (weighted) perceived bottled water as safer |
| Park 2019 | 65% perceived bottled water as safer |
| Park 2023 | 39% perceived bottled water as safer than tap.  In a 2023 study of US adults, those who believed bottled water is safer than tap were nearly six times as likely to drink more than 1 cup per day of bottled water than those who did not believe it is safer than tap (OR: 5.80; 95% CI: 4.67, 7.20). |
| Viscusi 2015 | 11% of those believing they had experienced a prior tap water induced illness and 8% who believed someone they knew experienced a prior tap water induced illness perceived bottled water as safer than tap. Discovered a relationship for US adults between ever being concerned about tap water safety and bottled water intake (RD: 0.1341; SE: 0.0346). Believing bottled water is safer than tap was also associated with an increased use of bottled water (RD: 0.2813; SE: 0.0287) |
| Gholson 2018 | 86-92% of those primarily using tap water thought it was safe compared to 57% of those who primarily purchased bottled water in their sample of 491 US adults. |
| Geerts, 2020 | In Belgium, Flemish adults with health and safety concerns about tap water consumed more bottled water than those without concerns (Standardized β: 0.152; p<0.001). |
| Regnier, 2015 | Respondents in a survey of households living on the Texas Mexico border reported significantly higher bottled water intake if they were concerned with tap water safety. |
| Dupont 2010 | In a survey of 1633 Canadian adults, average bottled water intake was significantly higher (52% vs 22%) among those who believed tap water caused serious problems than in the population as a whole. Among 1633 Canadian adults, the odds ratio for bottled water intake as the primary water source was 4.32 (95% CI: 1.92, 3.15) for those considering bottled water as safer than tap water and 2.46 (95% CI: 1.92, 3.15) for those who had a health concern with their tap water |
| Hu 2011 | In a survey of 5823 US adults, respondents who did not view home tap water as safe were 5.9 times as likely to exclusively use bottled water (OR: 5.88; 95% CI: 4.46, 7.76) and 1.7 times more likely to regularly use BW (OR: 1.74; 95% CI: 1.31, 2.32). |
| McLeod 2014 | In rural Saskatchewan, households who had ever had a prior water advisory had 70% higher odds of being primary BW drinker (OR: 1.7; 95% CI: 1.3, 2.4). The odds of using bottled water as the primary water source was also greater for households who believed their tap water was not safe, with a larger magnitude for those who had no aesthetic water complaint (OR: 2.3; 95% CI: 1.4, 3.8) compared to those with an aesthetic water complaint (OR: 8.5; 95% CI: 5.2, 13.9). |
| Johnstone 2012 | Discovered a stronger relationship in 10 OECD countries between negative perceptions about healthfulness of tap and the decision to purchase bottled water (OR: 14.22; SE: 1.10) |
| Bauer 2022 | In the US, frequency of bottled water intake was 64% higher for children whose mothers were unsure of tap safety (IRR: 1.64; 1.09, 2.45) (Bauer, 2022). |

### SSBs

| **Study** | **Finding** |
| --- | --- |
| Hess 2019 | Focus group participant explained that some people view the water as “not health” because it’s “dirty” which causes people to choose sweet drinks instead. |
| Scherzer 2010 | Latino parents living in the US reported that although they believe water is the healthiest beverage for their children, many will provide SSBs such as soda, Gatorade, or juice if bottled or filtered water are unavailable. |
| Sarkar 2015 | Some residents expressed concern about feeling like they have to choose between untreated water or high sugar drinks. SSBs were often more affordable than bottled water in the local store, so they were the more affordable option for meeting fluid needs. |
| Fledderjohnson, 2015 | Living in a household without clean water decreased the odds of Indian children (6-59 months) drinking any water during the prior day by 25% for well-water reliant households (OR: 0.75; 95% CI 0.64, 0.89) and by 30% for river, spring, or rainwater reliant households (OR 0.70; 95% CI 0.53, 0.92) compared to households that used piped, bottled, or tanked water. When children in the study population did not consume water, 24% consumed formula, other liquid, juice, or a combination of beverages. |
| Bauer, 2022 | Study of Medicaid insured US mothers found that although there was no association with perceived safety of tap water alone, mother or child SSB intake was associated with negative perceptions of tap water which considered safety and organoleptic properties. Each unit increase on the 5-unit negative tap water perception scale was associated with a 56% increase (IRR: 1.56; 99% CI: 1.18, 20.6) in weekly maternal SSB consumption and 54% increase (IRR: 1.54; 99% CI: 1.00, 2.36) in weekly child fruit drink consumption. Paradoxically, each unit increase on the 5-unit home tap water perception scale was associated with a 31% decrease in weekly child fruit drink consumption (IRR: 0.69; 99% CI: 0.49, 0.97). |
| Park 2023 | US adults who viewed tap water as unsafe or bottled water as safer than tap had a nonsignificant 12% (1.12; 0.86, 1.46) and significant 39% (1.39; 1.11, 1.75) higher odds respectively of daily SSB intake |
| Mosites, 2019 | Investigated the role of piped water in the home in SSB intake among Alaskans and found that the unadjusted odds of SSB intake was 46% higher in households without access to piped water (β: 1.46; 95% CI: 1.29, 1.67), however it lost statistical significance after adjusting for covariates (β: 1.29; 95% CI: 1.00, 1.67). |
| Wierda, 2022 | A study of Michigan adults before and after the Flint Water Crisis discovered that adults living in Flint, Michigan had a higher odds of soda (1.24; 95% CI: 1.01, 1.45), fruit juice (1.55; 95% CI: 1.14, 2.11), and other sugary drink (1.44; 95% CI: 1.04, 2.01) intake after the crisis compared to before |
| Onufrak 2012 | Mistrust of local tap water was associated with 2 times the odds of daily SSB intake for Hispanic adults (OR: 2.0; 95% CI: 1.2, 3.3) |
| Onufrak 2014 | Belief that school water fountains are unclean was associated with 2.9 times the odds of daily SSB intake for Hispanic students (OR: 2.9; 95% CI: 1.3, 6.6) |

## Taste

### Packaged Water

| **Study** | **Finding** |
| --- | --- |
| Collier 2020 | In a study of low socioeconomic North Carolina families seeking treatment for their children in obesity clinics, taste was the most common “sole reason” for exclusively drinking bottled water instead of tap (25.6%) and the most common partial reason (69.8%). This ranked even higher than perceived healthfulness, which was only the sole reason for exclusive bottled water intake for 16.1% of respondents and a partial reason for 60.3%. |
| Fortunato 2020 | In a 2020 study of two regions in Argentenia, the taste of bottled water was considered a valued quality of bottled water by more respondents than safety (34.4% vs 27.0%). Residents of high water-access areas were more likely to choose bottled water due to habit or taste preferences, while resident in low water access areas were more likely to value health/safety |
| Geerts 2020 | 15.2% of participants listed taste as the reason for drinking bottled water instead of tap. “Inferior taste” of tap water was associated with bottled water consumption (standard beta: 0.444; p<0.001). |
| Gungor-Demirci 2016 | Environmental engineering students who primarily used tap water listed taste as the least important characteristic while bottled water users listed it as the second most important characteristic. |
| Hoare 2014 | Disliking the taste of tap water was provided as a barrier to introducing infants to water. Mothers in the study who give their children bottled water listed concerning taste of tap water as a reason. |
| Spicer 2020 | Water taste had a larger impact on bottled water intake when at home (reported reason for 41-56% of males and 4-33% of females) than when on the land (reported reason for 0% of males and 3-7% of females). |
| Jones 2006 | Approximately 61% of respondents reported drinking bottled water in their home instead of the water from their private supply, with 69% of these individuals listing improved taste of bottled water as important or very important in this decision. |
| Dupont 2010 | Found a significant increase in the odds of primarily drinking BW associated with reporting an unpleasant tap water taste (OR: 2.90; SE: 1.25) |
| Aslani 2021 | 95.8% of respondents listed undesirable taste or hardness as a tap concern and a reason to choose bottled water instead. |
| Barrett 2017 | Taste was identified as one of the main drivers of beverage selection for youth who participated in the focus group. Bottled water was viewed as having a better taste than tap water, with one participant citing chlorine taste as the problem with tap water. |
| Queiroz 2013 | Among households in municipality C who only drink bottled water, bad taste, particularly chlorine, emerged as a central theme about tap water perceptions. |
| Scherzer 2010 | A majority of the community members avoided municipal tap water. Salty or strong chlorine taste were listed as reasons that residents believed the water was unsafe. The few respondents who believed local tap water was safe clarified that they still chose bottled water or water mill water over tap due to taste preferences. |
| Johnstone 2012 | The effect of disliking tap water taste was greater on BW purchasing (OR: 12.95; SE: 1.11) than home purification (OR: 3.62; SE: 1.12) in a survey of OCED households. |
| Park 2023 | For US adults, believing tap does not taste good was associated with nearly 3 times the odds of drinking more than 1 cup per day of bottled water (OR: 2.91; 95% CI: 2.39, 3.53). |

### SSBs

| **Study** | **Finding** |
| --- | --- |
| Sarkar 2015 | Sugary additions such as Kool-Aid or juice are sometimes used to improve the taste of water |
| Park 2023 | Perception of tap water taste was not related to SSB intake after controlling for covariates |

## Convenience/Accessibility

### Packaged Water

| **Study** | **Finding** |
| --- | --- |
| Geerts 2020 | In Belgium, a 2020 study of Flemish adults discovered that lack of potable tap water was significantly associated with bottled water usage (ρ: 0.152; p<0.001) |
| White 2013 | Studied variation in water source selection between the rainy and dry seasons in rural Cambodia, reporting that increased availability increased rainwater usage during the rainy season, but households with access to good roads were more likely to obtain distributed bottled water from the Teuk Saat 1001 non-governmental organization (NGO) year-round. |
| Patel 2011 | The pilot study, conducted in a US middle school, observed a significant increase in tap water intake, but no change in bottled water intake after providing filtered tap water dispensers and promotional activities, with 29% of students interviewed post-intervention saying that they preferred bottled water to tap water |
| Lawman 2020 | Installed new bottled water filling stations in Philadelphia recreation centers observed no change in the average number of youth bringing bottled waters, despite an average adjusted increase in tap water usage of 8.6 gallons per day per intervention site compared to the control sites (Difference in differences (DID): 8.6; 95% CI: 4.2, 13.0) |
| Grummon 2019 | The intervention targeting childcare centers (children aged 2-5 years old) that included increasing water provision and testing/remediation for lead observed no change in bottled water intake |
| Aslaniid 2021 | When surveyed about their rationale for using bottled water, 71.7% reported that purchasing bottled water was convenient. |
| Hess 2019 | In this qualitative study, parents of middle and high school students living in the rural south-western USA explained that bottled water is more convenient than tap water because it provides reliable water access even if students are in an area without clean tap water |
| Queiroz 2013 | Convenience was a central idea that emerged about bottled water, mentioned by 5 of the 30 interviewees. Convenience was attributed to packaging and the fact that unlike tap water, there is no need to clean filters or boil water. |
| Collier 2020 | Convenience was given as the sole reason for choosing bottled water over tap water by 7.5% of the study respondents. When providing multiple reasons, 47.2% listed convenience as a reason to select bottled water over tap. No significant difference was observed in the percent of White and non-White study participants who chose bottled water due to convenience (p=0.102). |
| Santillan-Vasquez 2022 | Despite increases in tap water intake after water filter provision, some participants still reported that bottled water is more convenient than tap water, particularly when leaving home. |
| Gungor-Demirci 2016 | A similar percent of tap and bottled water users listed convenience as the major reason for selecting their preferred water source, however more respondents consumed bottled water outside the home than inside the home which they most commonly attributed to convenience. |
| Scherzer 2010 | When at home, some participants drank filtered water instead of bottled water, however bottled water was the primary water source when not at home. |
| Mbagaya 2011 | 12.5% of respondents listed convenience as their reason for consuming bottled water. |
| Patel 2019 | No difference was observed in the number of community members with bottled water after this intervention which increased community water access, however authors noted that changes in bottled water usage was not a primary objective of the study. |
| Rosenthal 2023 | Installed water fountains in 10 San Fransisco parks, however a statistically significant reduction in visitors observed with bottled water or SSBs was not observed. |

### SSBs

| **Study** | **Finding** |
| --- | --- |
| Espinosa-Montero 2013 | Decisions about beverage intake were impacted by availability, and SSBs were more available than water in some worksites. |
| Rodriguez-Villamil 2013 | Bottled or bagged water were economically inaccessible for many households, resulting in soft drink purchases when residents were trying to conserve and not purchase water |
| Barrett 2017 | Youth included in the focus groups explained that water was often not available in cafeterias, rather milk and chocolate milk were the most commonly available beverages. |
| Kenney, 2019 | With insufficient access to water, school staff were hesitant to enforce sugar drink restrictions. |
| Loughridge 2005 | After the pilot study provided water coolers to two UK secondary schools, students still purchased similar levels of soft drinks, despite increasing water intake from the water coolers. |
| Patel 2011 | Although the pilot study observed a significant increase in tap water intake, there was no change in SSB intake after providing filtered tap water dispensers and promotional activities in US middle schools. |
| Lawman 2020 | After installing new bottled water filling stations in Philadelphia recreation centers, no change was observed in the average number of youth bringing SSBs despite an average adjusted increase in tap water usage of 8.6 gallons per day per intervention site compared to the control sites (Difference in differences (DID): 8.6; 95% CI: 4.2, 13.0). Among intervention center staff however, there was an 34.8 day reduction in the frequency of SSBs consumed over the past thirty days compared to the control center staff (DID: -34.8; 95% CI: -67.7, -1.9). |
| Grummon 2019 | The intervention targeted at childcare centers (children aged 2-5 years old) included increasing water provision and testing/remediation for lead and observed a decrease in intake of total “less-healthy” beverages (DID: -5.9 mL/d; 95% CI: -11.2, -0.6) compared to the control group |
| Irwin 2020 | Conducted a three-arm intervention in seventeen Ontario elementary schools that installed bottle fillers in all sites and additionally provided one of two educational programs at sites in two of the arms. None of the arms observed a significant change in SSB intake |
| Pinket, 2016 | Installation of water drinking stations and provision of education in schools in 6 European countries resulted in a significant decrease in prepacked fruit juice across all sites (-33 mL/d intervention, -10mL/d control; DID: -23.5 mL/d; p<0.001) |
| Kamin 2022 | The intervention provided and promoted water and non-sugar beverages instead of SSBs in four Slovenian private schools and reported decreases in intake of beverages w/added sugar, beverages with sugar, and sweet beverages. No significant change was observed for beverages with sweeteners and juice. |
| Visscher 2010 | The intervention placed water coolers in the canteens of 6 different Dutch secondary schools observed no reduction in SSB sales post-intervention |
| Hess 2019 | Parents of middle and high school students living in the rural south-western USA noted that SSBs were more convenient than water in some communities, resulting in higher intake |
| Zoellner 2012 | Focus groups with daily SSB consuming adults in rural southwest Virgina revealed that the availability of preferred water sources, whether tap or bottled, impacted decision to choose water or SSBs |
| Godin 2017 | Secondary school students in Guatemala attending public schools, which did not have access to water coolers, consumed soft drinks (Relative rate: 1.28; 95% CI: 1.16, 1.41)), sweetened coffee/tea (Relative rate: 2.00; 95% CI: 1.78, 2.24), and energy drinks (Relative rate: 3.32; 95% CI: 2.50, 4.40) more frequently than students attending private schools |
| Hipp 2015 | Studied how workplace water fountain availability impacted the beverage decisions of Missouri adults, and although there was not a significant relationship with overall SSB intake, the odds of soda intake was lower with higher water fountain availability (OR: 0.72; 95% CI: 0.58, 0.89) (Hipp, 2016). Surprisingly, water cooler presence was associated with higher frequency of SSB consumption (IRR: 1.21; 95%CI: 1.10, 1.32), especially sports drink consumption (IRR: 1.91; 95% CI: 1.43, 2.54). |
| Fehring 2019 | When water was made more available in three Aboriginal and Torres Strait Islander communities, including installation of chilled water bubblers and greater increased bottled water in stores, the percent of participants reporting consuming more water than sugary drinks due to availability of the two options increased from 11% to 44% in all studied communities. Additionally, sugary drink sales decreased by 3.4% and proportion of drink sales from SSB decreased 1.4% |
| Patel 2019 | No difference was observed in the number community members with sugar sweetened beverages in this intervention that increased community water access, however the authors noted that this outcome was not a primary objective of the study. |
| Rosenthal 2023 | Installed water fountains in 10 San Fransisco parks, however a statistically significant reduction in visitors observed with SSBs was not observed |

## Treatment/Filtering

### Packaged Water

| **Study** | **Finding** |
| --- | --- |
| McLeod 2014 | In rural Saskatchewan, respondents who did not treat their tap water were more likely to drink primarily bottled water than those who did not. Among those who use community water, the odds of someone who does not treat their water choosing primarily bottled water were 4.6 times of someone who does treat their water (OR: 4.6; 95% CI: 2.9, 7.3). The odds ratio decreases among those who do not use community water (OR: 2.5; 95% CI: 1.9, 3.3) |
| Rosinger 2018 | After adjusting for sociodemographic characteristic, US adults who did not use water treatment devices had 21% higher odds of drinking bottled water (OR: 1.21; 95% CI: 1.01, 1.4) |
| Reese 2022 | In a randomized control trial that provided low-income Latino parents of infants and toddlers with water filter pitchers, In the study arm that also received education, parents and children were consuming more water from tap water than from bottled water intake at the end of the intervention |
| Hocuk 2020 | 85% of households used bottled water prior to construction of a new drinking water treatment plant, and 83% still used bottled water after its construction. |
| Lee 2020 | Ecuadorian households decreased treatment of their drinking water after the local water treatment plant was upgraded, however they began to rely on bottled water instead of returning to treatment after the water treatment plant was damaged and became increasingly unreliable. |

### SSBs

| **Study** | **Finding** |
| --- | --- |
| Reese 2022 | The randomized control trial provided low-income Latino parents of infants and toddlers with water filter pitchers. SSB intake decreased by 11.2 fluid ounces/d (p<0.01) for parents and 1.50 fluid ounces/day (p=0.03) for children in the group that received a water filter pitcher and education, and it decreased by 8.0 fluid ounces/day (p<0.01) for parents and 1.6 fluid ounces/day (p=0.02) for children in the group that only received a filter. |
| Scherzer, 2010 | The majority of parents in the study reported that they will give their children sports drink or juice instead of tap water if no filtered or bottled water is available. |

## Cost

### Packaged Water

| **Study** | **Finding** |
| --- | --- |
| Rodriguez-Villamil 2013 | “Carrying water” is considered time, effort, and money intensive. Bottled/bagged water was too expensive for many participants, however the cost of boiling/treating rainwater was also prohibitively expensive for some. Some participants connect to the aqueduct, but they report quality concerns which officials attributed to “illegal connections”. |
| Patel 2010 | Cost of updating water fountains, filters, and providing maintenance seen as a large barrier to promoting tap water in schools. Providing free water viewed as potentially negatively impacting school finances through decreased beverage sales or concerns about potential vendor contract/USDA reimbursement violations. Bottled water considered too expensive by many, but still purchased even by low-income students due to concerns about tap water. |
| Loughridge 2005 | Students expressed discontent with feeling like they had to purchase bottled water instead of using free tap water in order to have “palatable water”. |
| Doegah 2018 | In the focus group of Ghanan teenagers and young adults, participants explained that the perception of bottled water brands as a status symbol has led to rapidly increasing costs. Purchasing bottled/sachet water was considered expensive, but one participant explained that the alternative was drinking no water if cold or bottled/sachet water couldn’t be afforded since the public water was considered unsafe. |
| Scherzer 2010 | Surveyed participants would not want to purchase water if their tap was proven to be safe. Some households reported that a 10-20 cent increase per gallon of water would cause financial hardship |
| Vasquez 2009 | No observed difference in willingness to pay (WTP) for an improved water system between Mexican households who buy bottled water and those who don't. Households spent an average of 3.55% of their monthly income on bottled water, which resulted in 7.49% of monthly income being spent on water when considering tap costs as well. |
| Larson 1999 | In 1999, Russian residents in Moscow spent >20,000 rubles per week on bottled water which was $3.50 in USD at the time (or around $6.50 in 2024). Boiling and settling water were considered “no-cost” water options and were consequentially regular water options for more survey respondents (88% and 33% respectively) than filtering (23%) or buying bottled water (13%). |
| Mbagaya 2011 | The estimated average percent of monthly income spent by 120 surveyed Kenyan consumers on bottled water was 9.6%. An association of bottled water with success and status also arose in a survey of Kenyan consumers, with 16.6% listing “fashion” as a consumption reason |
| Queiroz 2013 | Average % spent on bottled over tap water: 67%(municipality A), 31%(municipality C), N/A (municipality B). Percent average income spent on bottled water: 1% (municipality A), 3% municipality C, N/A (municipality B)  2 low-income participants in a study of Brazilian bottled water consumers, stating that they liked bottled water because it is a “fashion statement” |
| Geerts, 2020 | Bottled water similarly had a social element in a Belgian study where survey respondents preferred bottled water when visitors were present |

### SSBs

| **Study** | **Finding** |
| --- | --- |
| Hess et al., 2019 | Adult and student respondents agreed that water needed to be free to be preferentially selected over SSBs. School vending machines sold bottled water for more than 1 USD which was more expensive than most SSBs in the community |
| Patel 2010 | Two school stakeholders expressed concern that increasing intake of free tap water in schools could negatively impact school extracurricular activities which are funded by sales of alternative beverages. Other stakeholders in the school shared the misguided belief that serving water in cafeterias would prevent USDA reimbursement for school meals. |

## Appearance/Turbidity

### Packaged Water

| **Study** | **Finding** |
| --- | --- |
| Jones 2006 | Bottled water drinkers ranked reduced cloudiness as an important characteristic of bottled water |

### SSBs

| **Study** | **Finding** |
| --- | --- |
| Sarkar 2015 | Kool-Aid was promoted as a method of covering off-colors in tap water |

## Temperature

### Packaged Water

Discussed as a consideration when choosing water source, but not explicitly discussed in comparison between packaged and tap.

### SSBs

| **Study** | **Finding** |
| --- | --- |
| Hess 2019 | Rural Southwestern US middle school and high schooler stated that cold tap water was preferable than non-chilled soda |
| Thurber, 2020 | Australians surveyed were more likely to choose SSBs than warm water |

## Water Testing

### Packaged Water

| **Study** | **Finding** |
| --- | --- |
| Lavalle, 2021 | Private well using adults in Ontario who did not test their water had higher odds of primarily using bottled water than those who tested their well water (OR = 2.15; 95% CI 1.31–3.55) |
| Jones, 2006 | Many viewed bottled water as safer than tap water, a factor respondents attributed to “better testing” |
| Patel, 2019 | No changes in proportion of people drinking SSBs or bottles water (1.95% bottled water) post-intervention |
| Fortunato, 2020 | In a study testing nitrate concentration in bottled water in Argentenia, 8.7% of residents surveyed about their perceptions had bottled water quality concerns, but 82.4% still believed bottled was better for tap and 38.7% cited health as the reason for selecting bottled water. |
| Mbagaya 2011 | In a survey assessing reasons for bottled water consumption in Nairobi, Kenya, the study authors noted concerns that there are insufficient regulatory guidelines for bottled water in Kenya |
| Queiroz 2013 | In Brazil, Queiroz et al. (2013) noted that although there are national guidelines regulating bottled water, unauthorized retailers may not adhere to these guidelines |
| Lee 2020 | In Ecuador, Lee et al. (2020) found that diarrhea rates were not lower among bottled water users in their study population and noted that prior studies found high rates of contamination in bottled water in Ecuador. |
| Komarulzaman 2017 | Considered the switch from piped or commercial bottled water to often lower quality refillable bottled water in Indonesia concerning, noting that lower-income households were more likely to purchase these bottles due to the lower cost compared to commercial bottled water. |

### SSBs

| **Study** | **Finding** |
| --- | --- |
| Patel, 2019 | No changes in proportion of people drinking SSBs or bottles water (0.37% SSB) post-intervention |

## Hardness

### Packaged Water

| **Study** | **Finding** |
| --- | --- |
| Jones 2006 | Bottled water was considered superior to tap water due to its reduced hardness |

### SSBs

Not discussed

# Race/Ethnicity Reference Groups

## Packaged Water

| **Study** | **Finding** |
| --- | --- |
| Azlan, 2012 | No significant differences were found in tap water perception among survey respondents of different ethnicities (Malay, Chinese, Indian, Other), but significant differences were observed in bottled water perception with Indian respondents reporting the highest perceived quality and Chinese respondents reporting the lowest perceived quality. |
| Enzennia, 2023 | In the adjusted model of the association of school drinking water security experience on school water intake, Asian or Pacific Islander students drank bottled water 0.3 times per day more frequently while at school than White students (RD: 0.3; 95% CI: 0.05, 0.6). |
| Collier. 2020 | Higher exclusive bottled water intake was seen among non-White families compared to White families, even after stratifying by SES (low SES: 82.1% vs 57.5% p<0.001; high SES: 85.7% vs 45.2% p=0.001). |
| Drewnowski, 2013 | The lowest bottled water intake was observed among non-Hispanic White adults and the highest intake was observed among Mexican American adults. |
| Dupont, 2014 | Ontario First Nations communities were more likely to believe bottled water was safer than tap water when compared to cross-Canada non-First Nations survey respondents (OR 1.6; 95%CI: 1.38, 1.93). Ontario First Nations respondents were more likely to spend >$50 per month on bottled water (OR 4.9; 95%CI: 3.56, 6.83) and more likely to consume no tap water (be a 100% bottled water drinker) (OR 9.1; 95%CI: 6.91-12.12) than non-First Nations survey respondents. |
| Liu, 2023 | No variation in averting expenditures was seen when comparing Black, Hispanic, or Other race consumers to White consumers. |
| Newman, 2023 | 3/4 of Hispanic parents reported tap water was unsafe compared to 1/3 of non-Hispanic Black and non-Hispanic White parents. The majority of parents who believed tap water was unsafe purchased bottled or filtered water. |
| Onufrak, 2012 | More non-Hispanic Black (40%), Hispanic (34%), and non-Hispanic other (37%) reported that bottled water is safer than tap water than White respondents (22%) (p<0.001). |
| Regnier, 2015 | Individuals who considered themselves to be Hispanic reported drinking significantly more bottled water than those who did not. Bottled water intake was higher among residents of Mexico than among residents of the US. |
| Rosinger 2018 | Found higher odds of bottled water consumption for non-Hispanic Black (OR: 2.20; 95% CI: 1.79, 2.69) and Hispanic (OR: 2.37; 95% CI: 1.91, 2.94) adults than non-Hispanic white adults in the US. Furthermore, adults born outside the contiguous US (OR: 1.46, 95% CI: 1.19, 1.79) also had higher odds of consuming bottled water. |
| Park, 2019 | Belief that bottled water is safer than tap did not significantly vary by Hispanic heritage (p=0.88) or acculturation level (p=0.75). There was no significant variation in those who reported they would buy less bottled water if they new local tap water was safe by Hispanic heritage (p=0.34) or acculturation level (0.45). |
| Park, 2023 | Found that non-Hispanic Black and Hispanic adults in the US were more likely to believe bottled water is safe than tap water compared to non-Hispanic White adults (50% and 49% vs. 35%; p<0.001), however odds of bottled water intake were not found to have an association with race/ethnicity after adjusting for cofounders. |
| Viscusi, 2015 | Black (p<0.01) and not White or Black (p<0.05) respondents were more likely than White respondents to believe bottled water is safer than tap. |

## SSB

| **Study** | **Finding** |
| --- | --- |
| Hess, 2019 | One focus group participant stated that water was less accessible in local American Indian communities which resulted in higher SSB intake. |
| Mosites, 2020 | American Indian/Alaska native heritage (yes vs no) was associated with increased frequency of SSB intake (β=1.62; 95%CI: 1.05, 2.50) |
| Onufrak, 2014 | The odds of SSB intake were almost three times higher (OR: 2.9; 95% CI: 1.3, 6.6) for Hispanic participants who disagreed that school fountains were safe and clean compared to those who were neutral or agreed in a study of US students. An association between school tap water perceptions and SSB intake was not observed for students who identified as any other race/ethnicity. |
| Onufrak, 2012 | For US adults who believe bottled water is safer than tap water compared to those who do not, one study found higher prevalence of SSB intake for non-Hispanic respondents (47% vs 30%; p=0.01) but lower prevalence of SSB intake for non-Hispanic Black respondents (31.5% vs 43.1%; p=0.02). |
| Park, 2023 | There was no significant interaction observed between perceptions of tap water safety, bottled water safety, or tap water taste with SSB intake by race/ethnicity (Non-Hispanic White, Non-Hispanic Black, Hispanic, Non-Hispanic Other/Multi-Race) |
| Wierda, 2022 | Likelihood of drinking soda (OR: 1.8; 95%CI: 1.4, 2.3), fruit juice (OR 2.4; 95%CI: 1.9, 3.1) and other sweetened drinks (OR: 4.2; 95%CI: 3.2, 5.6) was higher for African American respondents than non-Hispanic White respondents in Zip codes affected vs not affected by the Flint Water Crisis. |
